# Simulation of single-protein nanopore sensing shows feasibility for whole-proteome identification

**DOI:** 10.1371/journal.pcbi.1007067

**Published:** 2019-05-30

**Authors:** Shilo Ohayon, Arik Girsault, Maisa Nasser, Shai Shen-Orr, Amit Meller

**Affiliations:** 1 Department of Biomedical Engineering, Technion–IIT, Haifa, Israel; 2 Rapport Faculty of Medicine, Technion–IIT, Haifa, Israel; 3 Department of Biomedical Engineering, Boston University, Boston, Massachusetts, United States of America; University of Illinois at Urbana-Champaign, UNITED STATES

## Abstract

Single-molecule techniques for protein sequencing are making headway towards single-cell proteomics and are projected to propel our understanding of cellular biology and disease. Yet, single cell proteomics presents a substantial unmet challenge due to the unavailability of protein amplification techniques, and the vast dynamic-range of protein expression in cells. Here, we describe and computationally investigate the feasibility of a novel approach for single-protein identification using tri-color fluorescence and plasmonic-nanopore devices. Comprehensive computer simulations of denatured protein translocation processes through the nanopores show that the tri-color fluorescence time-traces retain sufficient information to permit pattern-recognition algorithms to correctly identify the vast majority of proteins in the human proteome. Importantly, even when taking into account realistic experimental conditions, which restrict the spatial and temporal resolutions as well as the labeling efficiency, and add substantial noise, a deep-learning protein classifier achieves 97% whole-proteome accuracies. Applying our approach for protein datasets of clinical relevancy, such as the plasma proteome or cytokine panels, we obtain ~98% correct protein identification. This study suggests the feasibility of a method for accurate and high-throughput protein identification, which is highly versatile and applicable.

## Author summary

Macromolecules identification methods are central for most biological and biomedical studies, and while the field of genomics advanced to single-molecule resolution, the proteomic field still relies on bulk and costly techniques. We describe a solution for single protein identification, based on the analysis of optical traces obtained from fluorescently-labeled proteins threaded through a nanopore and processed by a pattern recognition algorithm. To evaluate the feasibility of our method we constructed computer simulations of the system, producing and analyzing nearly 10^8^ individual protein translocations from the human Swiss-Prot database. Our results suggest protein identification of >95% for the whole human proteome, even under non-ideal conditions. These results constitute the basis for a novel whole proteome identification method, with single molecule resolution.

## Introduction

Modern DNA sequencing techniques have revolutionized genomics [1], but extending these methods to routine proteome analysis, and specifically to single-cell proteomics, remains a global unmet challenge. This is attributed to the fundamental complexity of the proteome: protein expression level spans several orders of magnitude, from a single copy to tens of thousands of copies per cell; and the total number of proteins in each cell is staggering [2]. Given the lack of *in-vitro* protein amplification assays the ability to accurately quantify both abundant and rare proteins hinges on the development of single-protein identification methods that also feature extraordinary-high sensing throughput. To date, however, protein sequencing techniques, such as mass-spectrometry, have not reached single-molecule resolution, and rely on bulk averaging from hundreds of cells or more [3]. Affinity-based method can reach single protein sensitivity [4], but depend on limited repertoires of antibodies, thus severely hindering their applicability for proteome-wide analyses. Consequently, in the past few years single-molecule approaches for proteome analysis based on Edman degradation [5] or FRET [6] have been proposed. To date, however, profiling of the entire proteome of individual cells remains the ultimate challenge in proteomics [7].

Nanopores are single-molecule biosensors adapted for DNA sequencing, as well as other biosensing applications [8,9]. Recent nanopore studies extended nucleic-acid detection to proteins, demonstrating that ion current traces contain information about protein size, charge and structure [10–17]. However, to date, the challenge of deconvolving the electrical ion-current trace to determine the protein’s amino-acid sequence from the time-dependent electrical signal has remained elusive. In an analogy to the field of transcriptomics, in many practical cases it is sufficient to identify and quantify each protein among the repertoire of known proteins, instead of re-sequencing it. Yao and co-workers showed theoretically that most proteins in the human proteome database can be uniquely identified by the order of appearance of just two amino-acids, lysine and cysteine (K and C, respectively) [18]. But taking into account experimental errors, for example due to false calling of an amino-acid, or an unlabeled amino-acid, sharply reduces the ID accuracy. Motivated by recent experiments suggesting the ability to translocate SDS-denatured proteins through either small nanopores (~0.5 nm) [19], or large nanopores [20] (~10 nm), and the possibility to differentiate among polypeptides based on optical sensing in nanopore [21], we here introduce a protein ID method that according to simulation remains robust against the expected experimental errors. We show that relatively low-resolution, tri-color, optical fingerprints produced during the passage of proteins through a nanopore, preserve sufficient information to allow a deep-learning classification algorithm to accurately identify the entire human proteome with >95% accuracy. Even in cases where the apparent spatial and temporal resolutions of the optical system appear to be prohibitively low, and the amino-acids labelling efficiency is incomplete, whole proteome ID efficiency remains high and robust. Particularly, the expected protein ID efficiency is of an extremely high clinical relevancy. We illustrate the broad applicability of the method by analyzing the human plasma proteome, as well as commercially-available cytokine identification panel based on antibodies, showing that our antibody-free method can readily surpass current techniques in a number of key parameters, while displaying a near perfect accuracy.

## Results

### Simulation of nanopore-based recognition of proteins

**Fig 1:**
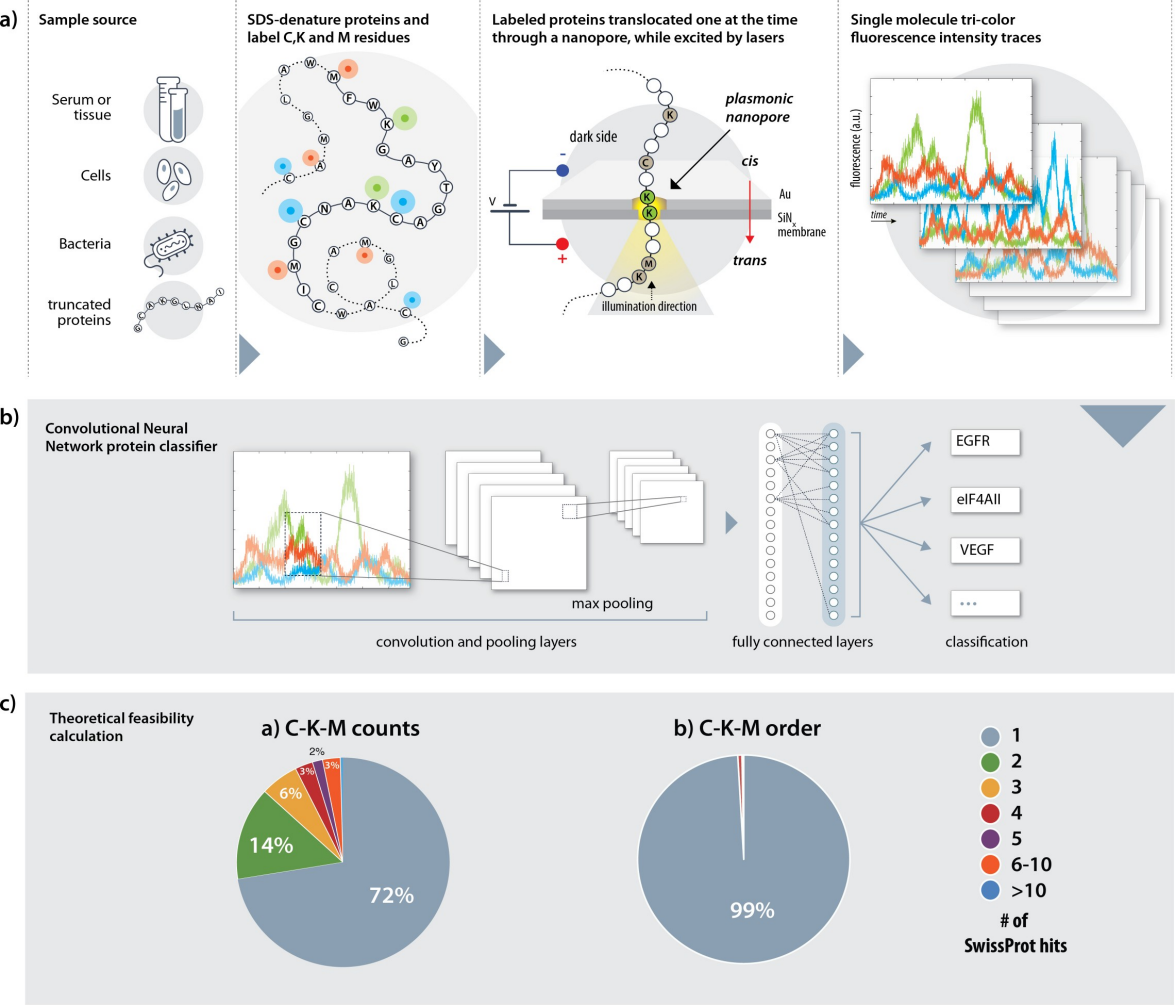
An overview of the Nanopore, tri-color protein identification method. **(a) A tentative sample process flow**. The protein sample is first denatured using SDS and cysteines (C), lysines (K) and methionines (M) are labeled with three spectrally-resolvable fluorophores. The labeled, SDS-denatured proteins are then threaded through a nanopore and excited by a laser light focused by a plasmonic architecture. The plasmonic field ensures local excitation of small portions of the denatured proteins. Finally, the photon emissions from each protein are measured in three channels, one for each fluorophore species, to create a tri-color optical trace per translocation. (b) A pre-trained convolutional neural network (CNN) classifier subsequently examines and classifies each trace, extracting its relevant features using a convolutional, an activation, a pooling and a fully connected layer, to identify the protein. (c) A theoretical evaluation of whole proteome fingerprinting based on complete labeling of C, K and M amino-acids. Counting only the number of labeled amino-acids yields unique identifications (ID) of 72% of all proteins. The remaining 28% of proteins were not uniquely identified and were either identified as one out of two (green slice) or more proteins as indicated by color. Considering also the order of the labeled amino-acids increases the unique ID fraction to 99%.

In our method, proteins extracted from any source (serum, tissue or cells), are denatured using urea and SDS (Fig 1A). Three amino-acids lysine (K), cysteine (C) and methionine (M) are labeled with three different fluorophores using three orthogonal chemistries: the primary-amines in lysines are targeted with NHS esters; thiols in cysteines are targeted with maleimide groups, and methionines are labeled using the two-step redox-activated chemical tagging [22]. The negatively charged SDS-denatured polypeptides are electrophoretically threaded, one at a time, through a sub-5 nanometer pore fabricated in a thin insulating membrane to ensure single file threading of the SDS-coated polypeptide. The voltage, nanopore diameter and other factors, such as solution viscosity are used to regulate the protein translocations speed. The nanopore is illuminated using laser beams for multi-color excitation [23]. The excitation volume (Fig 1A, yellow highlighted region) is centered with the nanopore, and importantly, its axial depth is confined by plasmonic focusing of the incident electromagnetic field [24]. Consequently, depending on the excitation depth, either a single or multiple labeled amino-acids will be simultaneously illuminated, during the passage of the protein. Three-color fluorescence time traces (“fingerprints”) are recorded for each protein passage and are classified using deep-learning (Fig 1B).

The theoretical likelihood of protein ID can be tested by calculating the percentages of unique matches of all proteins in the human Swiss-Prot database [25] based on the number and the order of appearance of three amino-acids only. Simply counting the number of K, C and M residues in each protein identifies 72% of the total proteins uniquely, and another 14% identified as either one of two proteins in which one of them is the correct match (online methods). Moreover, the percentage of uniquely identified proteins is close to 99% with the determination of the KCM order of appearance along all proteins in the human proteome database (Fig 1C). Thus, in principle, the boundaries for the expected ID accuracies fundamentally permit whole-proteome, single-protein, identification.

The theoretical analysis shown in Fig 1C may be considered as an upper limit for the accuracy of a protein ID method based on a three amino-acid labelling, which neglects inter-dye distances. However, it ignores experimental limitations, such as the sensing spatial and temporal constraints, the labelling efficiency and the photophysical properties of fluorophores. These factors are likely to impact the accuracy of the protein ID method, and hence must be considered. To this end we developed a detailed photophysical model to numerically calculate the time-dependent photon emission during the passage of each SDS-denatured protein through a solid-state nanopore. Our model consists of three layers: first, we used Finite Difference Time Domain (FDTD) computations to evaluate the expected electromagnetic field distribution for a simple plasmonic structure fabricated on top of the nanopore (Materials and Methods). Second, an amino-acid labelling simulation was applied to each protein, in order to generate partial labelling of each of the three target amino-acids. Finally, SDS-denatured proteins were allowed to slide through the plasmonic nanopore complex while illuminated at three distinct wavelengths. The expected detected photon emissions were calculated at each step of the protein translocation taking into account the photophysical properties of the fluorophores, as well as energy transfer (FRET), bleaching kinetics and collection efficiencies. This allowed us to generate detailed photon emission time traces for each and every protein translocation.

**Fig 2:**
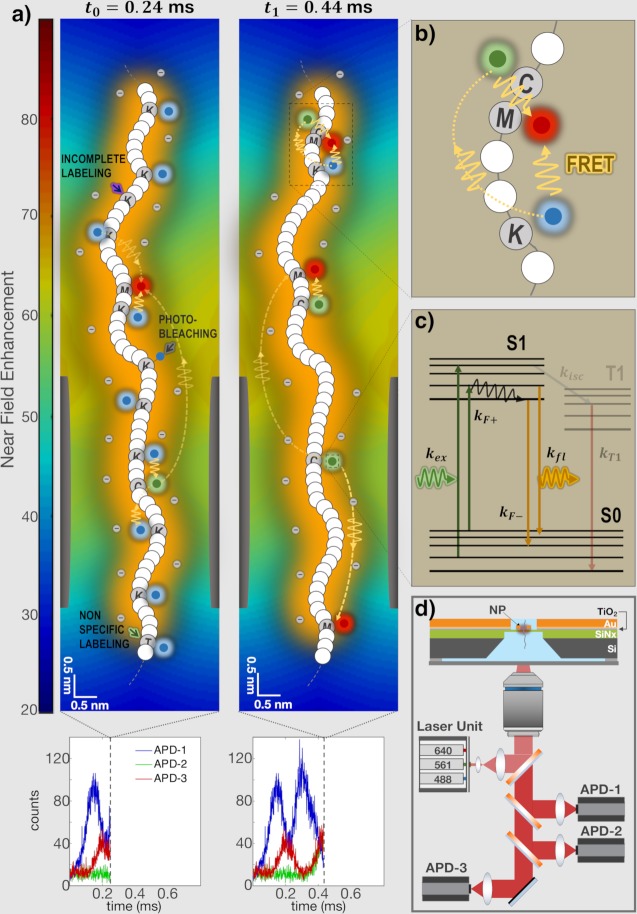
Simulation of the fluorescence signals generated during the translocation of the SDS-denatured PH and SEC7 domain-containing (PSD) protein. (a) The nanopore diameter and height were set to 3 and 5 nm, respectively, and the plasmonic architecture deposited on its ‘top-side’ produced a confined excitation profile (14–20 nm axial full-width half maximum) whose color map displayed on the left indicates the excitation near field enhancement at a wavelength of 640 nm (modeled using FDTD, see S1 Note). Two snapshots of the translocation process are shown and denoted by the timepoints *t*_*0*_ and *t*_*1*_ at which they were respectively taken. Energy transfer, photo-bleaching, incomplete labeling and non-specific labeling are indicated by dotted yellow lines, solid grey, purple and green arrows, respectively. (b) Zoomed in region of the polypeptide in which Forster resonance energy transfer (FRET) is shown in greater details. In this configuration, energy was transferred from lysine fluorophores to cysteine and methionine emitters, and from cysteine to methionine fluorophores. (c) The fluorescence emission rate of each labeled amino-acid was modeled as either a two-state or three-state system (see online methods for further details and in which *k*_*F+*_ and *k*_*F-*_ refer to *k*_*FRET*,*+*_ and *k*_*FRET*,*-*_, respectively). *k*_*exc*_ denotes the absorption rate, *k*_*isc*_ the inter-system crossing rate and *k*_*T1*_ the triplet state relaxation rate. Fluorophores are depicted in a color which denote the excitation wavelength with which they are excited or the channel to which they belong. (d) Schematics of the nanopore chip and optical system, which includes a high NA water immersion objective lens, three excitation laser lines and corresponding APDs. The nanopore chip is made of four consecutive layers: silicon (grey), silicon nitride (green) in which the nanopore is drilled, titanium oxide (grey blue) and gold (orange).

To illustrate our method, we schematically show in Fig 2A snap-shots of the system at two time points during the passage of the PSD protein. This figure is plotted in scale to illustrate the relative dimensions of the plasmonic field, the nanopore and the SDS-coated polypeptide chain (marked as orange layer around the chain). Specifically, the axial FWHM of the plasmonic field is 20 nm calculated from the FDTD field distribution, and the nanopore diameter is 3 nm. Each protein was modeled as a fully-denatured, SDS-coated, wormlike polymer [26], translocating across the nanopore at an instantaneous velocity *u*_*i*_ = 〈*u*〉+*δu*_*i*_ where 〈*u*〉 is its average velocity, and the random term *δu*_*i*_ accounts for thermal fluctuations in its motion. Since the SDS-coated biopolymers have a Kuhn length of approximately 7 nm [26], they can be assumed to be partially-stretched (unfolded) wormlike polymers during translocation through a sub ~5 nm pore. Moreover, when threaded through a 3 nm pore, the roughly 2 nm wide SDS-coated proteins are confined laterally in a small volume in the nanopore proximity where the electromagnetic field remains nearly constant. Hence, in this study the protein translocations can be treated as one dimensional [27]. The excitation profile calculated from the FDTD simulations was approximated by a one-dimensional Gaussian function as shown in S1 Fig. The fluorescence emission rate of each labeled amino-acid while passing through the excitation zone was modeled as a two-state system (Fig 2C), as described in the Materials and Methods section. Triplet state transition rates, which may result in microsecond-long dark-states were also investigated (equations not shown) based on literature values of three specific fluorophores [28–30]. We explicitly took into consideration energy transfer rates (Fig 2B and 2C), which directly depend on the amino-acid sequence, as well as photo-bleaching rates (indicated by dotted yellow lines and solid grey arrows in Fig 2, respectively). At each time step of the simulation the emitted light from all fluorophores residing in the excitation zone were split to three spectrally-resolved, photon-counter channels as shown in Fig 2D. In addition to the collection and detection efficiency of each channel, we also considered photon statistics by incorporating shot-noise.

The labeling efficiency was modeled by randomly positioning fluorophores at the K, C and M amino-acid, such that in each protein only a fraction Γ_*j*_ of them (*j* represents K, C or M) was actually labelled (indicated by purple arrows in Fig 2A). In all the following computational results presented the three amino-acids, K, C and M were labelled by Atto488, Atto565 and Atto647N fluorophores, and the fluorophores properties were taken into account when simulating the photon emission rates. Additionally, we introduced cross-labelling efficiency (green arrows in Fig 2A), although this is known to be negligible [31].

**Fig 3:**
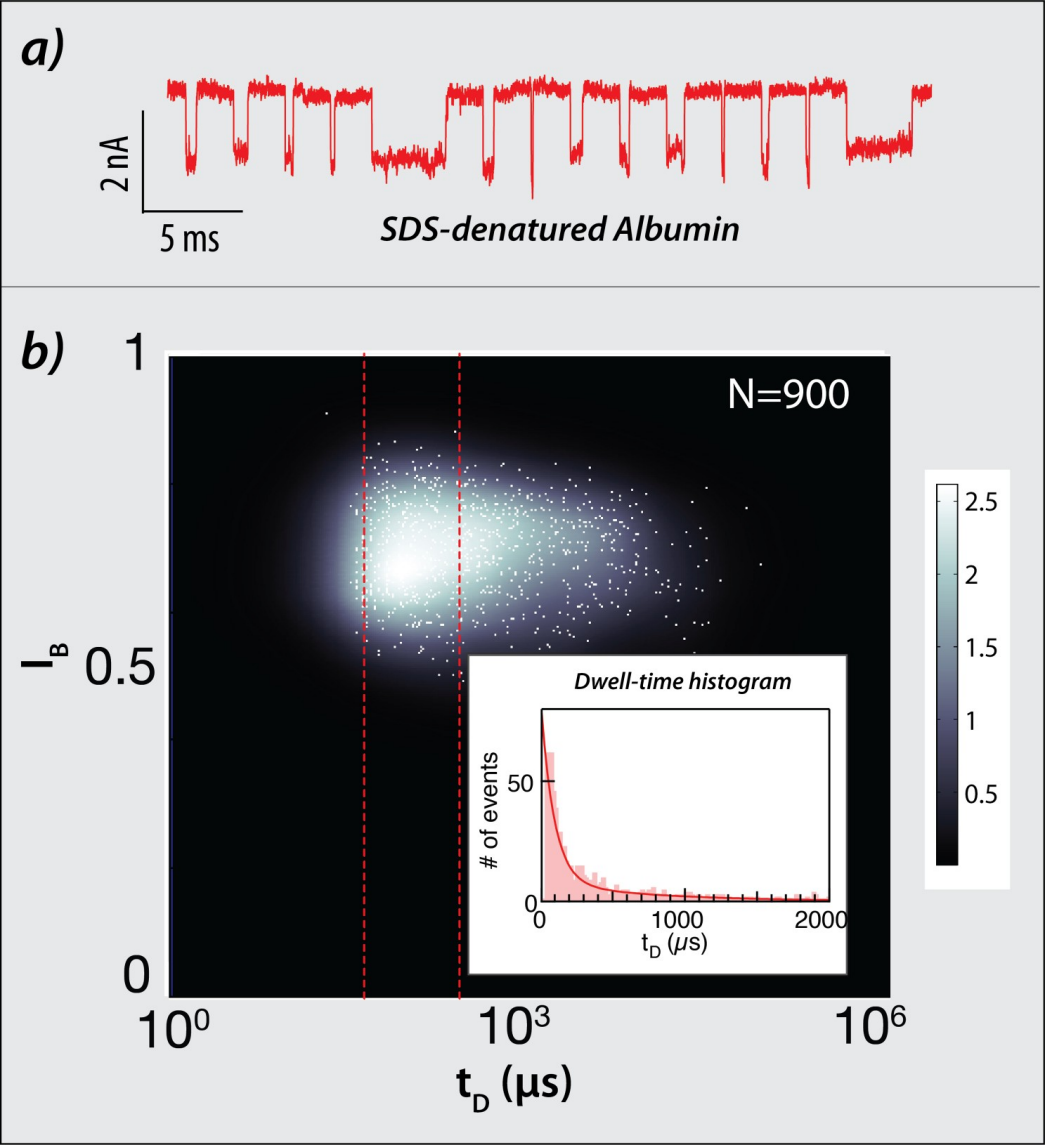
Measurements of SDS-denatured human serum albumin translocations through solid-state nanopores. (a) Electrical events of albumin translocating through a 4 nm-wide nanopore measured at 300 mV. (b) Scatter plot of the fractional blockade current I_B_ versus the translocation time t, with its corresponding density map. The number of translocations events displayed amounts to 900. The inset shows the dwell-time histogram, fitted to an exponential decay with characteristic time of 94.3±7.2 μs.

In order to estimate the translocation velocity of SDS-denatured polypeptides we performed electrical translocation measurements using SDS-denatured albumin (585 amino-acids) proteins using ~4 nm-wide solid-state nanopores, as described in the Materials and Methods section. Representative translocation events measured at a bias voltage of V = 300 mV, in which a single blockage current level is observed, are shown in Fig 3A. Examining a statistical set of >900 translocation events showed a single blockade current level (*I*_*B*_ = 0.7) indicative of single-file polypeptide translocations. This experiment supports the assumption that proteins are likely to be fully denatured as they thread through the narrow nanopore, in agreement with a previous publication [20]. Fig 3B displays an overlay of the scatter plot of the fractional blockade current *I*_*B*_ versus the translocation dwell-time *t*_*D*_, with its corresponding density map. The area delimited by the dashed red lines approximate the typical full-width-half-maximum of a Gaussian centered on the characteristic dwell-time (94.3±7.2 μs as determined by the histogram shown in the inlet panel). Accordingly, we estimate the mean translocation velocity by 0.2 cm/s. Notably, this velocity is slower than the previous report, presumably due to the fact that in our experiments a much smaller nanopore was used.

**Fig 4:**
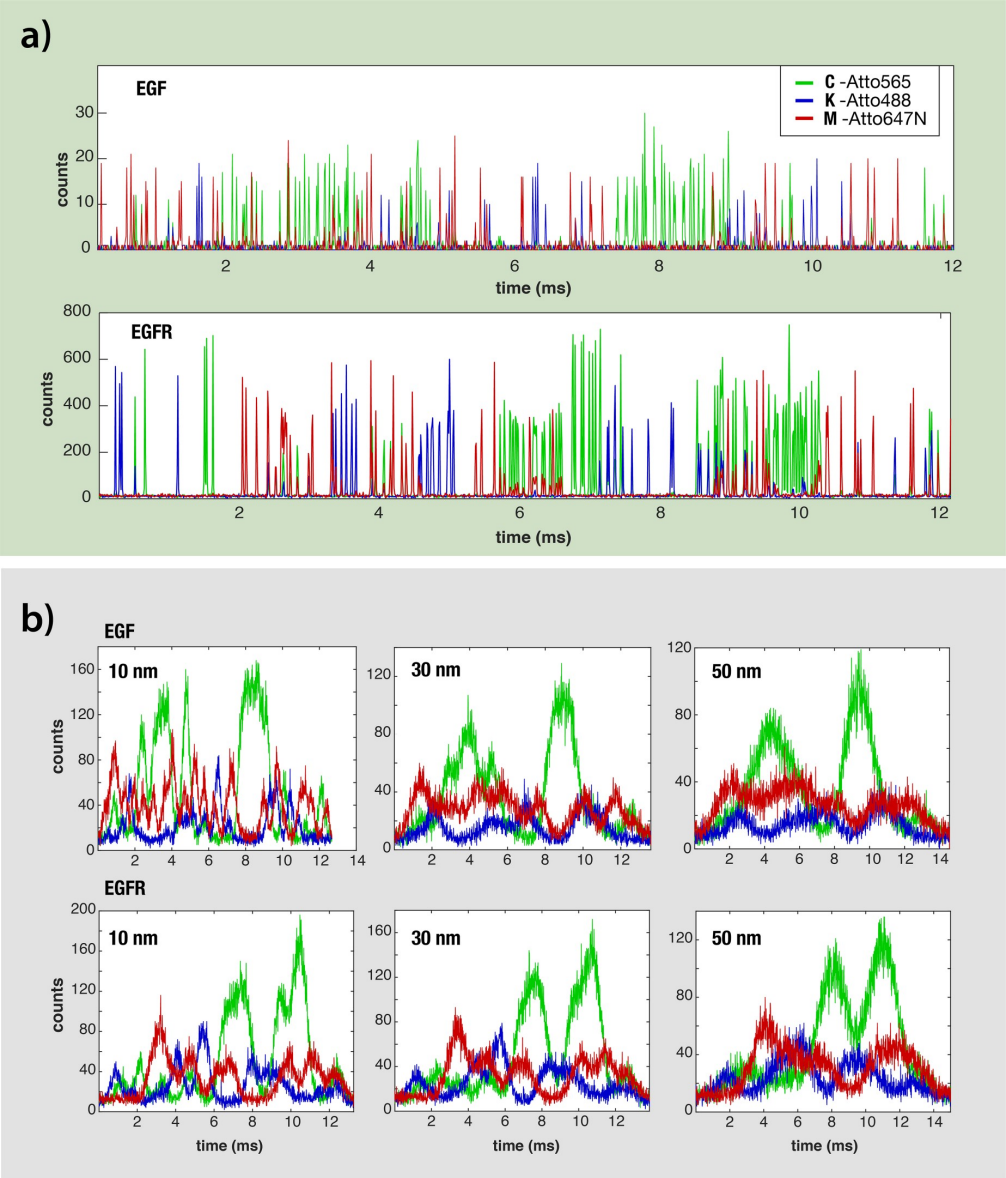
Simulated optical traces of epidermal growth factor (EGF) precursor protein and its receptor EGFR produced under different conditions. The C, K and M amino-acids were labeled using three different fluorophores as indicated. (a) Optical signals simulated using a spatial resolution of 0.5 nm and a labelling efficiency of 100%. (b) optical signals simulated using three distinct spatial resolutions: 10, 30 and 50 nm (from left to right).

We first focus on the simulated optical signals calculated for two proteins having nearly the same length: the EGF precursor, and its receptor EGFR (1208 and 1210 amino-acids, respectively). Under near-ideal experimental conditions (100% labelling, 0.5 nm resolution, and velocity of 0.035 cm/s) their tri-color fingerprints were readily distinguishable from each other, despite similar K, C and M compositions, and followed the actual K,C,M amino-acid order in each protein (Fig 4A). We then extended our protein translocation simulations under much lower spatial resolutions, lower labelling efficiencies and higher translocation velocities. As expected, in the more realistic conditions we no longer can resolve individual fluorophore photon bursts, associated to single K, C or M residues. Instead, the resulting signals appear as continuous tri-color fingerprints of each protein translocation. Importantly, however, the fingerprints, even at the poorest resolution of 50 nm maintain an overall pattern characteristic of each protein (Fig 4B). Analyzing >5·10^7^ single protein translocations events, under different conditions suggest that even at 100 nm resolution some characteristic features of each protein are preserved (S2 Fig). Moreover, we expect that small variations in the nanopore size would result in different translocation velocities. To evaluate this effect, we repeated the translocation simulation experiments at mean values of 0.035, 0.2 and 2 cm/s and increasing the translocation velocity fluctuations (20%, 30% and 40% of the mean velocity). Our result presented in (S3, S4 & S8 Figs) suggest that as long as the velocity is in the order of ~ 0.2 cm/s (or below) in accordance with our experimental result (Fig 3), the identification accuracy remains sufficiently high.

**Fig 5:**
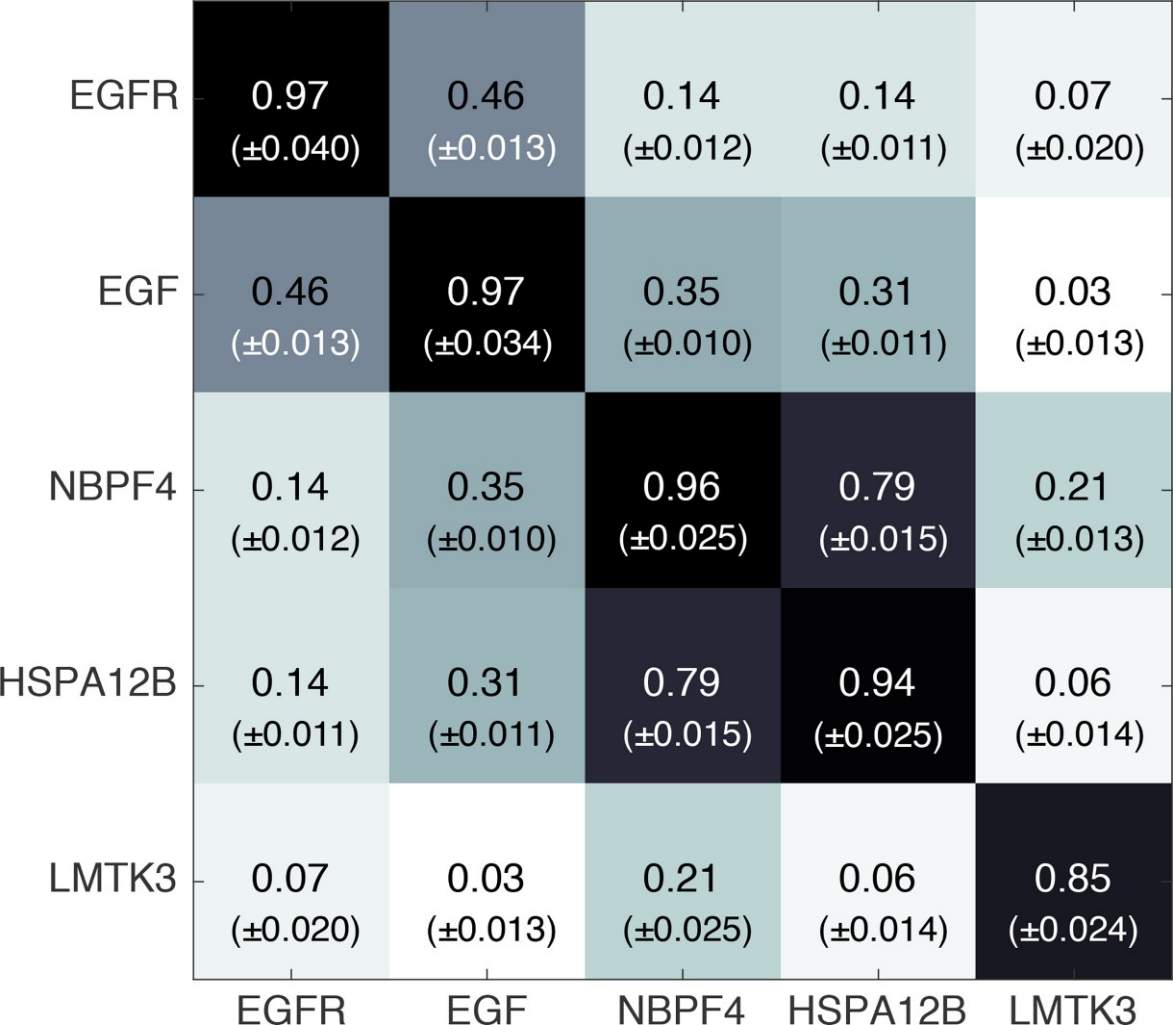
Pearson correlation among pairs of five simulated proteins photon traces. The elements of the correlation matrix, consisting of all Pearson correlation coefficients between all pairs of 50 translocation repeats, were first transformed to Fisher’s z, subsequently averaged and finally transformed back into an “average” Pearson correlation coefficient [32]. The standard deviation is given in parentheses.

We tested the similarity among repeated translocations of the same proteins, which were subject to different labeling and random velocity fluctuations, by evaluating the Pearson correlation coefficients between all pairs of 50 translocation repeats of the same protein. The results, showed in all cases high values (0.85–0.97) when considering auto-correlation (Fig 5, diagonal values). In contrast, attempting to cross-correlate among 5 different, randomly-chosen, proteins produced in most cases much lower Pearson coefficient values (0.03–0.35). Obviously, this is just a small fraction of all possible cross-correlations. However, even as is, this sample of data suggests that the protein translocation simulator generates highly-reproducible signals.

### Whole-proteome protein ID using deep-learning classification

**Fig 6:**
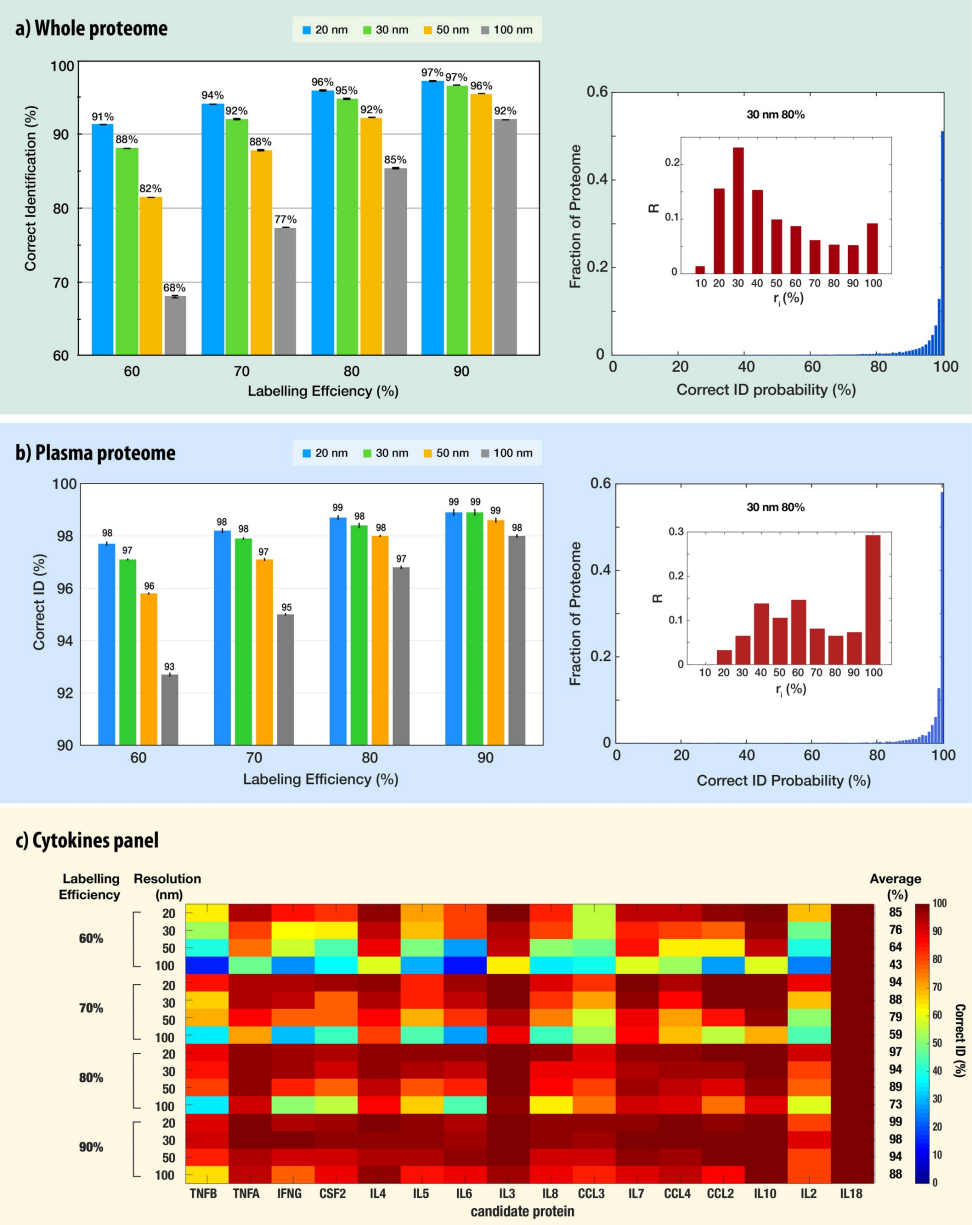
**CNN-based classification results of: a) whole proteome, b) plasma proteome, and c) a cytokine panel.** The fractions of the correctly-identified translocation events from whole-proteome classifications repeated five times are shown in a) and b) left panels. Each classification consisted of five separate training-and-testing of a CNN using 100 translocation events per protein (a total of ~10^7^ events), whose resulting correct identifications were averaged. These experiments and analyses were performed under four different spatial resolutions (20, 30, 50 and 100 nm) and labelling efficiencies (60, 70, 80 and 90%). Right-hand panels show the fraction of the proteome correctly identified with probability *p* when considering a spatial resolution of 30 nm for different labeling efficiencies. The bin size was set to 1%. The insets display the degree of randomness in misclassification. The bin height is given by the fraction of mis-identified proteins *R* (i.e. proteins that had at least 10% of their events misclassified) at different *r*_*i*_ (fraction of identical mismatch) intervals: *r*_*i*_ = max_*j*_*n*_*ij*_/*N*_*i*_ for each protein *i*, where *n*_*ij*_ is the number of translocation events misidentified to protein *j* and *N*_*i*_ the total number of misclassified translocation events. The bin width–*r*_*i*_ interval size–was set to 10%. Other experimental conditions are provided in supporting information file. c) Cytokines panel identification using the same proteins as in the ELISA set “CytokineMAP A”. The heat-map represents the correct ID of each cytokine under the specified labelling efficiency and resolution. The average correct ID is provided in the right-hand column.

Next we vastly scaled-up our simulations to include thousands of different proteins, each one repeated hundreds of times under different labeling efficiencies, translocation velocities and spatial resolutions. The accurate classification of noisy, low-resolution, time-dependent signals is often encountered in areas such as image and speech recognition and is effectively handled by Convolutional Neural Networks (CNN) approaches [33,34]. We postulated that provided sufficient training, the CNN would be able to identify most proteins based on the tri-color fingerprints. To check this hypothesis, we set up deep-learning whole-proteome analyses. First, we trained the CNN network using a large data set containing at least 80 individual nanopore passages of each protein in the Swiss-Prot database. Then the CNN was presented with new protein translocation events and queried as to the protein identity. This procedure was repeated at least 5 times for whole-proteome analysis allowing us to establish the mean ID accuracy and its standard deviation, for 16 different experimental conditions (Fig 6A). Starting with the highest labelling efficiency (90%, right-hand set) we observed that 96%-97% of all protein translocations were correctly identified, as long as the spatial resolution was ≤50nm. The correctly identified protein fraction dropped down to 92% using a 100 nm resolution. A similar pattern can be observed for the other labelling efficiencies with somewhat lower numbers. In the worst-case scenario considered here (100 nm resolution and only 60% labeling efficiency) the CNN nevertheless was able to correctly classify 68% of all translocation events, similar to the ideal case considered in Fig 1C, (C, K, M counts only). In other words, despite the fact that 40% of the target amino-acids were not labeled, and the resolution of the probing was about a third of the optical diffraction limit, the pattern recognition algorithm identified correctly nearly 70% of all protein translocation events. When the labelling efficiency was improved to the expected standards (between 70%-90%) [22,35], and the sensing resolution assumed to be in the 20–30 nm, the correct identification of all translocation was roughly 95%. Increasing the translocation speed of proteins by nearly two orders of magnitude to 2 cm/s (an order of magnitude higher than the mean measured velocity in Fig 3), reduced the ID accuracy (S8 Fig). However, for high labeling efficiencies (80% and 90%) the ID accuracy was high (72% and 81%, respectively).

In addition to the mean accuracies, the CNN algorithm produces a “confusion matrix”, which presents the number of times each and every protein *x* was identified as protein *y* (where *x* and *y* could be any of the proteins in the set). We used this information to calculate the probability density function (pdf) of correct ID for each and every classification set, namely the likelihood that a given protein is correctly identified with probability *p*. The pdf of correct ID calculated for the case of 30 nm resolution and 80% labelling efficiency (Fig 6A right panel) indicates that 51%, 71% and 89.2% of proteins were correctly identified with probability of 1.0, 0.98–1.0 and 0.9–1.0, respectively. The probability distributions for all other conditions are shown in SI S5 and S6 Figs.

We also analyzed the results for misclassified proteins. Specifically, we were interested to know whether a misclassified protein is likely to be deterministically or randomly misclassified. To investigate the degree of randomness in misclassification, we first selected proteins that had at least 10% misclassified events. Then, we determined the fraction of identical mismatch *r*_*i*_ = max_*j*_*n*_*ij*_/*N*_*i*_ for each protein *i*, where *n*_*ij*_ is the number of translocation events misidentified to protein *j* and *N*_*i*_ the total number of misclassified translocation events. With this a high *r*_*i*_ was characteristic of a deterministic misidentification, i.e. protein *i* is consistently mistaken with another specific protein *j*, and conversely a low *r*_*i*_ was indicative of a rather random misidentification. As shown in the right panel of Fig 6A, proteins were often confused with several others, suggesting a relatively high degree of randomness in misclassification, while only 10% were consistently mis-identified, that is with the same partner. The distributions for all other conditions are shown in SI S5 and S7 Figs.

### Identification of plasma proteome and cytokines panels

We further evaluated the performance of our approach for clinically-relevant applications including whole human plasma proteome and a cytokine panel. In both studies, we kept the CNN training at the whole human proteome, rather than restricting it to the clinical sub-set. Then we presented nanopore translocation traces of the plasma/cytokines proteins and evaluated the classification accuracy as before. Interestingly for the high-spatial resolutions (20 nm and 30 nm) the correct ID of the 3852 plasma proteins was only slightly larger than the whole proteome accuracy at the different labelling efficiencies, reflecting the fact that there is a small set of proteins that are hard to be classified in both cases (Fig 6A and 6B right panel). However, at the lower resolutions, especially for the 100 nm case in which we observed a significant drop in the ID accuracy for the whole proteome results, we still obtained very high scores for the plasma proteome. Even at the lowest labelling efficiency of 60% at 100 nm resolution the CNN classified correctly 93% of all translocations (Fig 6B). In addition, the fraction of proteins correctly identified with probability between 0.9–1.0 improved over that of the whole-proteome classification, reaching 96.8% for the case of 30nm resolution and 80% labeling efficiency. Finally, close to 30% of mis-identified proteins were consistently mistaken with another specific partner, suggesting that the accuracy of classification could be further significantly improved by relaxing the requirements of correct ID for selected proteins. These results indicate that single-molecule plasma proteome application, which holds great clinical value, does not require extremely-stringent experimental resolutions or super-efficient labelling chemistries (S9–S11 Figs).

The cytokine panel (CytokineMAP [36]) contains 16 proteins involved in inflammation, immune response and repair. We evaluated the CNN classification under 16 different experimental conditions (Fig 6C). At the lowest labelling efficiency of 60% the ID accuracy drops between 43% - 85%, and at the realistic 80% labelling we obtain correct ID in the range of 73% - 97%. However, despite the functional similarity between the candidate cytokines, and the wide range of conditions tested, each was distinguishable from all other cytokines within the commercial test panel. This indicates that our approach has the potential to meet the requirements of a broad range of clinically relevant applications–that are less demanding than whole-proteome identification–with extremely high accuracies and yet very poor experimental conditions (S12 Fig).

## Discussion

Single-molecule protein ID and quantification techniques are on the verge of revolutionizing the field of proteomics by enabling researches to achieve single-cell proteomics and to identify low abundance proteins that are essential biomarkers in biomedical and clinical research [7]. Specifically, nanopore discrimination among poly-peptides based solely on two color labeling of C and K residues has recently been demonstrated [21]. Here, we have proposed and simulated the feasibility and limits of a novel method for single-molecule protein ID and quantification using tri-color amino-acid tags and a plasmonic nanopore device. Specifically, we designed a simulator that incorporates a range of physical phenomena to predict and model the behavior of our proposed device and performed a computational analysis taking into account a broad range of experimental conditions to characterize its performance. Importantly, we developed a whole-proteome single-molecule identification algorithm based on convolutional neural networks providing high accuracies (>90% overall), reaching up to 95–97% in challenging but attainable experimental conditions. To facilitate the computational efforts, in this study we approximated each protein translocation dwell-time using a Gaussian distribution function. Notably, past studies [37] successfully utilized CNN to identify signals from exponentially-distributed time-dependent signals, which may better reflect the experimental dwell-time distribution (Fig 3). However, further studies will be required to evaluate the full impact of the temporal distributions of proteins translocation dwell-time on the CNN identification accuracy.

In clinical samples lysine residues may be post-translationally modified hence reducing their labelling efficiency. To account for this effect and for the limitations in the chemical labelling yield, we evaluated the protein identification accuracy under partial labelling conditions. Our results (Fig 6) show that our tri-color protein identification method nevertheless largely circumvents this potential issue, yielding very high accuracies for up to 40% of unlabeled residues. This is attributed to a redundancy in the tri-color labelling scheme that provides a higher degree of robustness against partial labelling.

Solid-state nanopores can process tens of individual proteins per second, and importantly because our method does not rely exclusively on measurements of the ion-current through the pore, it lends itself for parallel readout of high-density nanopore arrays fabricated on a sub mm^2^ membranes, using multi-pixel single-photon sensors [38]. The versatility and robustness of convolutional neural networks tremendously simplify any calibration procedures and even potentially allow protein ID based on partial reads [39]. This ensures that the whole-proteome ID is reliable and compatible with a wide variety of systems, able to overcome real experimental challenges. Furthermore, in many cases (notably for the plasma proteome) misidentified proteins were consistently confused with another specific protein, which in a broad range of applications such as identifying disease-specific biomarkers, may not pose a significant issue as only small-subsets of the proteome are considered, or since the quantification of proteins can be cross-examined with expected counts (e.g. low, medium or high abundance). Finally, we evaluated the expected efficacy of our approach with commercially available applications, even resolving functionally similar proteins in rather poor experimental conditions.

## Methods

### A theoretical analysis of the proteins ID based on 2 or 3 amino-acid tags

The theoretical identification values were calculated using the human proteome Swiss-Prot database, which contains 20,328 entries. For each entry we extracted the number of the target amino-acids (C, K and M), as well as their order of appearance. For example, the p53 protein would either be characterized by its C,K,M counts (10, 20, 12, respectively) or by the sequence below: MKMMMKKCKMCKCMKMCCCMCCMMCCKKKKKKMKKKKKKKMK, in which all intervening amino-acids were deleted. Proteins having identical characteristic sequences (or C, K and M counts) are grouped together. A protein is identified when it is the sole member of a group. In the case of p53, both the C, K and M counts and the characteristic sequence gave a unique identification. The pie charts (Fig 1C) distribute the proteins according to the size of the group in which they belong to.

### A protein labeler program

Each protein primary sequence was transformed into a string (*B*(*i*)) to which we assigned a value of 1, 2 or 3 corresponding to each of the three *aa* tags (K, C, and M), respectively; and 0 for all other *aa* in the protein sequence. To account for partial or nonspecific labelling a set of randomly selected labeled positions in the string were omitted according to a given labeling efficiency (*η*_*L*_), and a set of artificial labeled positions were inserted according to a given nonspecific labeling efficiency (*η*_*NS*_). It is important to note that nonspecific labeling did not affect all *aa* equally. For instance, in generating a barcode for lysine (K) positions, nonspecific labeling could only be inserted at positions of either threonine, serine and tyrosine (amino-acids which have been shown to compete with NHS-ester-based labeling) with a probability of typically 1% [31]. The strings were generated for the entire Swiss-Prot data base, and were re-generated each time to simulate an uneven labelling of the same protein data sets, as well as whenever we used different values of *η*_*L*_ and *η*_*NS*_.

### Finite difference time domain calculation of plasmonic fields

The three-dimensional near field enhancement of the plasmonic structure (2D vertical cross-section shown in Fig 2A) was determined using a finite difference time domain (FDTD) [40] method solving for Maxwell’s time-dependent electromagnetic equations. The architecture over which the FDTD computations were performed comprised a 10 nm-thick silicon (Si) membrane–exhibiting a 3 nm-wide nanopore–on top of which a gold (Au) plasmonic structure was deposited (Fig 2D). An additional 2 nm-thick titanium oxide (TiO_2_) adhesive layer was inserted in between the Au structures and underlying Si membrane. The plasmonic structure consisted of a gold ring (inner and outer diameter of 12 and 32 nm, respectively, and a height of 40 nm) centered at the nanopore and embedded inside a gold nanowell (diameter of 120 nm and a height of 100 nm). Water was used as the immersion media.

The excitation field was modeled as a total-field scattering-field source (TFSFS) [41] and the spatial sampling frequency was set to 5 nm^-1^ (taking 60 frequency points over the 500–800 nm wavelength range). The FDTD boundary conditions consisted of 8-layer PMLs (perfectly matched layers) symmetric in the x axis and antisymmetric in the y axis thus minimizing the reflections and the computational cost, respectively. Frequency domain power monitors only were incorporated in the simulation to determine the near field enhancement in the vicinity of the nanopore. All numerical simulations were performed using Lumerical FDTD Solutions (Lumerical, Inc).

### Simulation of nanopore-based optical sensing of proteins

To simulate the translocation of the linearized protein through the nanopore, we assumed a unidirectional motion with steps of a single *aa* length (Δ≈0.35 nm) and an average velocity *u* (cm/s). To account for thermal fluctuations in this process we added a random noise term *δu* at each step (*δu* can be positive or negative). Hence the simulation step time of the *i*-th *aa* was defined as *τ*_*i*_ *=* Δ / (*u + δu*). The average protein velocity value was typically ~0.2 cm/s, based on experiments using SDS denatured proteins in solid-state nanopores as shown in Fig 3. Additionally, we tested faster translocations (2 cm/s). The fluorescence emission rate of each fluorophore *n* in our system *K*_*fl*,*j*,*n*_(*t*) was modeled as a two-state system:

$$K_{fl,j,n}(t)=k_{fl,j}P_{j,n}(t)$$ Eq 1

where *j* = 1..3 correspond to each of the three excitation/emission channels, *k*_*fl*_ the fluorescence transition rate and *P*_*n*_(*t*) the occupation probability of the excited molecular state *S*1. The fluorophores are excited by up to three laser lines corresponding to the three channels, that form sub-wavelength excitation volumes by means of a plasmonic nanostructure or total internal reflection. The axial full width at half maximum of our Gaussian excitation volume *I*_*ex*_ is defined as *ξ* and is allowed to vary from 5 nm to 200 nm in order to account for broad possible experimental conditions. The emitted light from the three-color channels is assumed to be acquired with given efficiencies *η*_*j*_, which include both the optical transmission efficiencies and the photodetector efficiencies. The photon counts $I_{i}^{j}$ at each channel *j* during each step *i* of the protein translocation is then determined by summing the emissions of all the fluorophores *n* that resides within the excitation volume. Namely:

$$I_{i}^{j}=\eta_{j}\sum_{n}K_{fl,j,n}(t_{i})+k_{bg}\tau_{i}=\eta_{j}\sum_{n}k_{fl,j}P_{j,n}(t_{i})+k_{bg}\tau_{i}$$ Eq 2

$$\left\{ \begin{array}{c} P_{j,n}(t_{i})=P_{j,n}(t_{i-1})+(\frac{k_{ex,j}(n)}{k_{j}(n)}-P_{j,n}(t_{i-1}))(1-e^{-k_{j}(n)t_{i}})\\ k_{j}(n)=k_{ex,j}(n)+k_{S1,j}=\sigma_{ex,j}\frac{I_{ex,j}(n)\lambda_{ex,j}}{hc_{0}}+\tau_{S1,j}^{-1} \end{array}\right.$$ Eq 3, Eq 4

where *k*_*bg*_ is the background emission rate, *t*_*i*_ the time at which step the translocation occurred such that *t*_*i*_*−t*_*i-1*_ = *τ*_*i*_, *k*_*ex*,*j*_(*n*) is the excitation rate of the fluorophore *n* of channel *j*, *σ*_*ex*,*j*_ is its absorption coefficient, *λ*_*ex*,*j*_ is the excitation wavelength and *τ*_*S1*,*j*_ is its excited state lifetime.

The number of cycles (S0→S1→S0) undergone by each fluorophore was capped to account for photobleaching according to a decaying exponential distribution. Specifically, the maximum number of cycles performed by each fluorophore before photobleaching was given by a random number drawn from a decaying exponential distribution with a characteristic decay of ~10^6^. Finally, we applied a Poisson distribution to the photon counts $I_{i}^{j}$ to simulate shot noise.

To include energy transfer (such as Förster Energy Transfer and homo-transfer) in our system we calculated a 2D distance matrix for each fluorophore in our system. The distances between the labelled *aa’s* (or fluorophores) in each linearized protein were subsequently used to calculate the Förster energy transfers of each fluorophore from and to each of its neighboring emitters. As a proxy for the exact energy transfer, two additional transition rates accounting for energy gain and loss were incorporated in the fluorophore two-state model:

$$\left\{ \begin{array}{c} k_{FRET+,j}(n)=\frac{1}{hc_{0}}\sum_{i}\sum_{m\neq n}\sigma_{ex,i}I_{ex,i}(m){E_{n\leftarrow m}\lambda }_{ex,i}\\ k_{FRET-,j}(n)=\frac{\sigma_{ex,j}I_{ex,j}(n)\lambda_{ex,j}}{hc_{0}}\sum_{i}\sum_{m\neq n}E_{m\leftarrow n} \end{array}\right.$$ Eq 5, Eq 6

where *E*_*m*←*n*_ = (1+(|*x*_*m*_−*x*_*n*_|/*R*_0,*m*←*n*_)^6^)^−1^ is the FRET energy transfer efficiency from fluorophore *n* to *m*, *x*_*n*_ is the position of fluorophore *n* along the denatured protein and R_0,m←n_ is the Förster-radius of the (*n*,*m*) dye pair when considering an energy transfer from fluorophore *n* to *m*. The transition rates *k*_*ex*,*j*_(*n*) and *k*_*j*_(*n*) in Eq 4 were corrected to account for FRET accordingly:

$$\left\{ \begin{array}{c} k_{ex,j}(n)\to k_{ex,j}(n)+k_{FRET+,j}(n)\\ k_{j}(n)\to k_{j}(n)+k_{FRET+,j}(n)+k_{FRET-,j}(n) \end{array}\right.$$

The code was implemented using MATLAB, and the optical readouts of the three channels were determined by running this procedure for each labeling string.

### Protein classification and mapping of optical reads to protein IDs

For the purpose of a multi-class (the human proteome comprises more than twenty thousand proteins) classification of time-series that exhibit specific patterns, we used convolutional neural networks (CNN) that have shown great promise in the field of pattern recognition, including image classification, which similarly requires tens of thousands of classes [42,43]. Specifically, we used the python deep learning package Keras on a four GPU architecture (NVIDIA Tesla K40), which leads to a CNN whole-proteome training time of ~2 h only. The CNN model relied on four sequential layers–a convolutional layer, a normalization layer in which dropout was applied and a pooling layer–followed by a multi-layer perceptron. In brief, the convolutional layer filters (at a given step or stride size) the translocation time-series with a large set of kernels of a specific size. The resulting activation or feature map it provides is further transformed by the normalization layer such that the mean and standard deviation of the activation map approach zero and one, respectively. Next, the dropout circumvents overfitting of the CNN to the training dataset by setting a random subset of activations to zero. The last pooling layer performs a down-sampling operation on the activation map to further prevent overfitting of the training dataset and the computational load. The multi-layer perceptron consists of a single densely-connected neural network layer, each neuron outputting the probability of belonging to the class it represents (‘softmax’ activation function).

The hyper-parameters were optimized according to standard procedures, that is maximizing the accuracy of the CNN trained over five to ten epochs per hyper-parameter set. Once finely adjusted, the CNN was trained using twenty epochs to yield the greatest accuracy. The protein identification accuracy as determined by the CNN was calculated as the fraction of correctly classified translocation events from the test dataset. We partitioned randomly the dataset into five pairs of training and testing sub-sets, and for which we determined the identification accuracy. The final accuracy was calculated as the average between them where a typical test set included ~400,000 translocation events.

### SDS-denatured protein translocation experiments

Solid-state nanopores were fabricated using a laser drilling method in 17 nm-thick SiN_x_ membranes as described previously [44]. Human serum albumin (Biological Industries Inc. 30-O595-A) was first treated by TCEP (5 mM) at room temperature for 30 min to break disulfide bonds and subsequently denatured at 90°C for 5 min in PBS with 2% sodium-dodecyl sulfate (SDS). The resulting albumin concentration was further diluted (100:1) to <1 nM in buffer (PBS/0.4M NaCl/ 0.1% SDS/ 1mM EDTA) for nanopore translocation experiments performed under a 300 mW bias. A custom-made LabVIEW interface was used to acquire and analyze each event. Scatter plots and dwell-time distributions were generated using Igor Pro (Wavemetrics).

## Supporting information

S1 Note All the numerical simulations were performed using Lumerical FDTD Solutions (Lumerical, Inc), solving for Maxwell’s equations using a finite-difference time-domain method. Additional information regarding the FDTD simulations can be found in the Supporting Information.
(DOCX)

S1 Fig Simulated optical traces of different proteins with or without a fluorophore triplet state. The spatial resolution and labeling efficiency were fixed in all cases to 30nm and 100%, respectively. Left column shows the simulated optical traces using a two-state (ground and excited) fluorophore model; right column using a three-state (ground, excited and triplet) model. Transition rates in between all states were determined according to the manufacturer (when available) and to published work (see Article).
(TIFF)

S2 Fig Simulated optical traces of the epidermal growth factor (EGF) precursor protein and its receptor EGFR generated using two spatial resolutions: 100 and 150nm (from left to right). The labeling efficiency was set to 100% and the average translocation velocity to 0.0035 *cm*/s.
(TIFF)

S3 Fig Simulated optical traces of the epidermal growth factor (EGF) precursor protein in different experimental conditions. (a) optical signals simulated using a spatial resolution of 0.5nm and a labelling efficiency of 100%. (b) optical signals simulated using three distinct spatial resolutions: 10, 30 and 50nm (top), three distinct labeling efficiencies: 90%, 80% and 70% (middle), three velocity fluctuations: 20%, 30% and 40% of the mean translocation velocity *v* = 0.035 *cm*/s (bottom).
(TIFF)

S4 Fig Simulated optical traces of the B Double Prime 1 (BDP1) protein in different experimental conditions. (a) optical signals simulated using a spatial resolution of 0.5nm and a labelling efficiency of 100%. (b) optical signals simulated using three distinct spatial resolutions: 10, 30 and 50nm (top), three distinct labeling efficiencies: 90%, 80% and 70% (middle), three velocity fluctuations: 20%, 30% and 40% of the mean translocation velocity *v* = 0.035 *cm*/s (bottom).
(TIFF)

S5 Fig Whole-proteome probability density function of correct identification and degree of randomness in misclassification at 30nm. The fraction of the whole proteome that was correctly identified with probability *p* (a) and the degree of randomness in misclassification (b) were determined for 30nm and four labeling efficiencies (60, 70, and 90%; the remaining 80% as well as the CNN accuracy bar plot are shown in the article). The bin size was set to 1% in all histograms. The bin height of histograms in (b) is given by the fraction of mis-identified proteins R (i.e. proteins that had at least 10% of their events misclassified) at different *r*_*i*_ (fraction of identical mismatch) intervals: *r*_*i*_ = max_*j*_*n*_*ij*_/*N*_*i*_ for each protein *i*, where *n*_*ij*_ is the number of translocation events misidentified to protein *j* and *N*_*i*_ the total number of mis-classified translocation events. High is characteristic of a low degree of randomness, and vice-versa low of a high degree of randomness. The bin width–*r*_*i*_ interval size–was set to 10%. The value in parentheses indicate the percentage of mis-identified proteins of a whole-proteome experiment.
(TIFF)

S6 Fig Whole-proteome probability density function of correct identification for different experimental conditions. The fraction of the proteome that was correctly identified with probability *p* was determined for three spatial resolutions (20, 50 and 100nm; 30nm shown in article) and four labeling efficiencies (60, 70, 80 and 90%). The bin size was set to 1% in all histograms.
(TIFF)

S7 Fig Whole-proteome degree of randomness in misclassification for different experimental conditions. The bin height is given by the fraction of mis-identified proteins R (i.e. proteins that had at least 10% of their events misclassified) at different *r*_*i*_ (fraction of identical mismatch) intervals: *r*_*i*_ = max_*j*_*n*_*ij*_/*N*_*i*_ for each protein *i*, where *n*_*ij*_ is the number of translocation events misidentified to protein *j* and *N*_*i*_ the total number of mis-classified translocation events. High is characteristic of a low degree of randomness, and vice-versa low of a high degree of randomness. The bin width–*r*_*i*_ interval size–was set to 10%. The value in parentheses indicate the percentage of mis-identified proteins of a whole-proteome experiment. The degree of randomness in misclassification was determined for three spatial resolutions (20, 50 and 100nm; 30nm shown in article) and four labeling efficiencies (60, 70, 80 and 90%).
(TIFF)

S8 Fig Whole-proteome protein identification accuracy as a function of amino-acid dwell time and labelling efficiency. The spatial resolution was fixed to 30 nm and the dwell-time was defined as the time it took a peptide to translocate over the length of a single amino-acid. The corresponding translocation velocities are 2, 0.2 and 0.035 cm/s. The APD binning was set to 1 *μ*s. The CNN classification was still robust to low labeling efficiency and realistic spatial and temporal resolutions, expected in real experiments.
(TIF)

S9 Fig Plasma-proteome probability density function of correct identification and degree of randomness in misclassification at 30nm. The fraction of the plasma proteome that was correctly identified with probability *p* (a) and the degree of randomness in misclassification (b) were determined for 30nm and four labeling efficiencies (60, 70, and 90%; the remaining 80% as well as the CNN accuracy bar plot are shown in the article). The bin size was set to 1% in all histograms. The bin height of histograms in (b) is given by the fraction of mis-identified proteins R (i.e. proteins that had at least 10% of their events misclassified) at different *r*_*i*_ (fraction of identical mismatch) intervals: *r*_*i*_ = max_*j*_*n*_*ij*_/*N*_*i*_ for each protein *i*, where *n*_*ij*_ is the number of translocation events misidentified to protein *j* and *N*_*i*_ the total number of mis-classified translocation events. High is characteristic of a low degree of randomness, and vice-versa low of a high degree of randomness. The bin width–*r*_*i*_ interval size–was set to 10%. The value in parentheses indicate the percentage of mis-identified proteins of a plasma-proteome experiment.
(TIFF)

S10 Fig Plasma-proteome probability density function of correct identification for different experimental conditions. The fraction of the plasma proteome that was correctly identified with probability *p* was determined for three spatial resolutions (20, 50 and 100nm; 30nm shown in article) and four labeling efficiencies (60, 70, 80 and 90%). The bin size was set to 1% in all histograms.
(TIFF)

S11 Fig Plasma-proteome degree of randomness in misclassification for different experimental conditions. The bin height is given by the fraction of mis-identified proteins R (i.e. proteins that had at least 10% of their events misclassified) at different *r*_*i*_ (fraction of identical mismatch) intervals: *r*_*i*_ = max_*j*_*n*_*ij*_/*N*_*i*_ for each protein *i*, where *n*_*ij*_ is the number of translocation events misidentified to protein *j* and *N*_*i*_ the total number of mis-classified translocation events. High is characteristic of a low degree of randomness, and vice-versa low of a high degree of randomness. The bin width–*r*_*i*_ interval size–was set to 10%. The value in parentheses indicate the percentage of mis-identified proteins of a plasma-proteome experiment. The degree of randomness in misclassification was determined for three spatial resolutions (20, 50 and 100nm; 30nm shown in article) and four labeling efficiencies (60, 70, 80 and 90%).
(TIFF)

S12 Fig Identification of proteins targeted by different commercial ELISA sets. a) Whole-proteome CNN accuracy of the CytokineMAP B kit proteins for four spatial resolutions (20, 30, 50 and 100nm) and four labeling efficiencies (60, 70, 80 and 90%). b) Whole-proteome CNN accuracy of the MetabolicMAP kit proteins for four spatial resolutions (20, 30, 50 and 100nm) and four labeling efficiencies (60, 70, 80 and 90%). c) Whole-proteome CNN accuracy of the NeuroMAP A kit proteins and misclassification distribution for four spatial resolutions (20, 30, 50 and 100nm) and four labeling efficiencies (60, 70, 80 and 90%).
(TIFF)

S13 Fig Simulated optical traces of different proteins with or without a fluorophore triplet state. The spatial resolution and labeling efficiency were fixed in all cases to 30nm and 100%, respectively. Left column shows the simulated traces optical traces using a two-state (ground and excited) fluorophore model; right column using a three-state (ground, excited and triplet) model. Transition rates in between all states were determined according to the manufacturer (when available) and to published work (see Article).
(TIFF)
