# Supplementary material for: Simulation of single-protein nanopore sensing shows feasibility for whole-proteome identification
Source: PLoS Comput Biol. 2019 May 30;15(5):e1007067. doi: 10.1371/journal.pcbi.1007067 (PMC6559672; doi:10.1371/journal.pcbi.1007067)
Supplement: S1 Note — Additional information regarding the FDTD simulations can be found in the Supporting Information. (DOCX) [file pcbi.1007067.s001.docx]

**Supplementary Note 1**

**Supplementary Note | FDTD Simulation**.

All the numerical simulations were performed using Lumerical FDTD Solutions (Lumerical, Inc), solving for Maxwell’s equations using a finite-difference time-domain method. The refractive index of SiNx and TiO2 was taken as 2.1 and 2.6, respectively. The optical constants of gold were taken from Johnson & Christy and those of water from Palik [1,2]. A uniform mesh size of 2 nm was first used. Mesh sizes of 0.5 and 1 nm were subsequently applied to a confined volume centered at the nanopore (6 x 6 x 20 nm^3^) and to a hollow region delimited by the nanopore volume and the gold nanowell, respectively, to resolve the field enhancement of nanometer-sized structures.

A TSFS (total field scattered field) source was modeled to illuminate the structure with a pulsed and vertically polarized plane wave of amplitude 1 V/m and wavelength range from 500 to 800 nm. The FDTD boundary conditions consisted of 8-layer PMLs (perfectly matched layers). Furthermore, the inherent symmetry of the plasmonic structures and the light polarization were used to apply anti-symmetric (horizontal x axis) and symmetric (horizontal y axis) boundary conditions to reduce the computational cost. The optical power of the near fields was determined using power monitors in the frequency domain, and were normalized to the optical power measured without any plasmonic gold structures. All unspecified parameters were set to default.

**References**

1. Johnson PB, Christy RW. Optical constants of the noble metals. Phys Rev B. 1972;

2. Palik ED. Handbook of Optical Constants of Solids [Internet]. Handbook of Optical Constants of Solids. Elsevier; 1985. Available from: https://linkinghub.elsevier.com/retrieve/pii/C20090209202
