# Supplementary figures and images for: Simulation of single-protein nanopore sensing shows feasibility for whole-proteome identification
Source: PLoS Comput Biol. 2019 May 30;15(5):e1007067. doi: 10.1371/journal.pcbi.1007067 (PMC6559672; doi:10.1371/journal.pcbi.1007067)

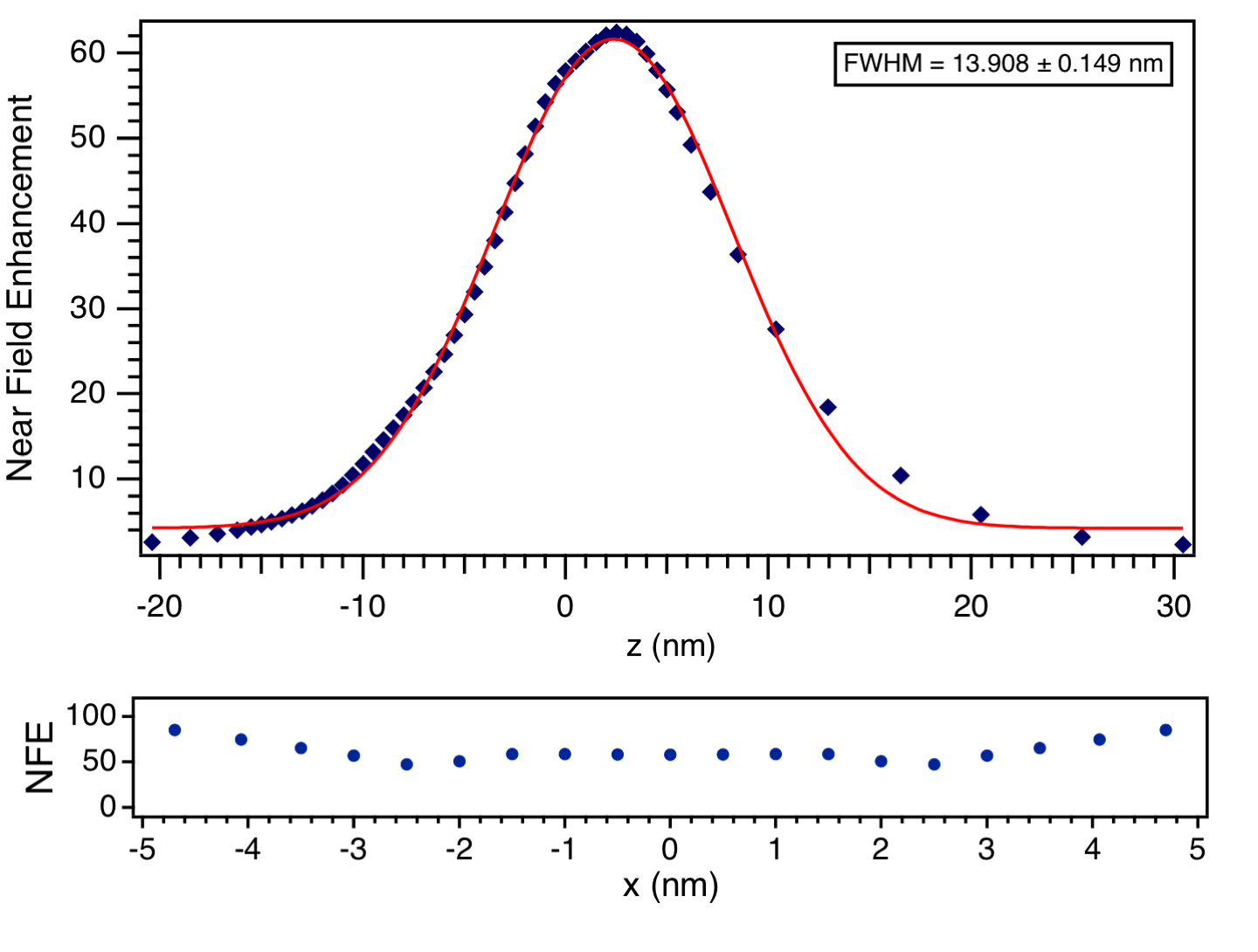

Supplement: S1 Fig — The spatial resolution and labeling efficiency were fixed in all cases to 30nm and 100%, respectively. Left column shows the simulated optical traces using a two-state (ground and excited) fluorophore model; right column using a three-state (ground, excited and triplet) model. Transition rates in between all states were determined according to the manufacturer (when available) and to published work (see Article). (TIFF) [file pcbi.1007067.s002.tiff]

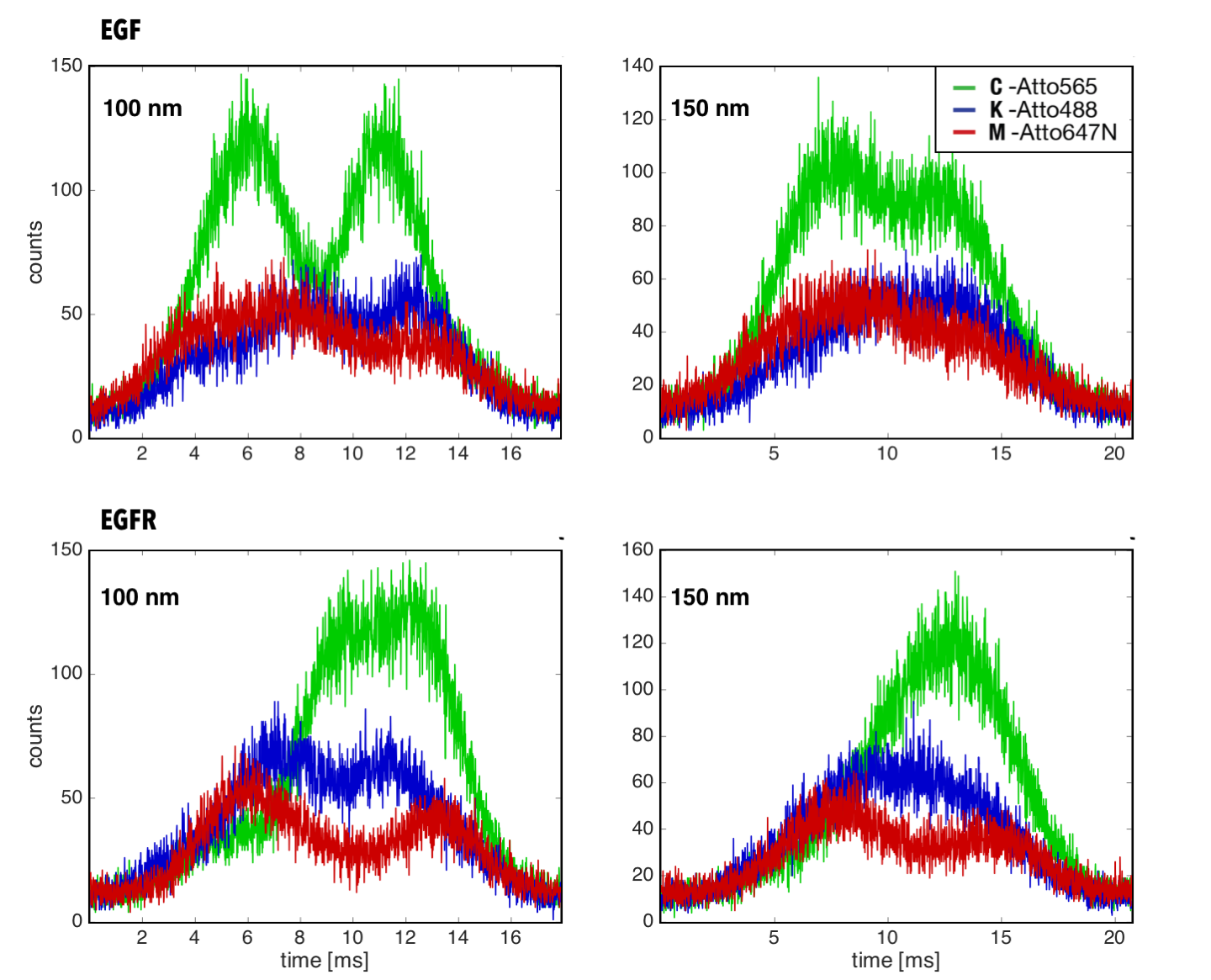

Supplement: S2 Fig — The labeling efficiency was set to 100% and the average translocation velocity to 0.0035 cm/s. (TIFF) [file pcbi.1007067.s003.tiff]

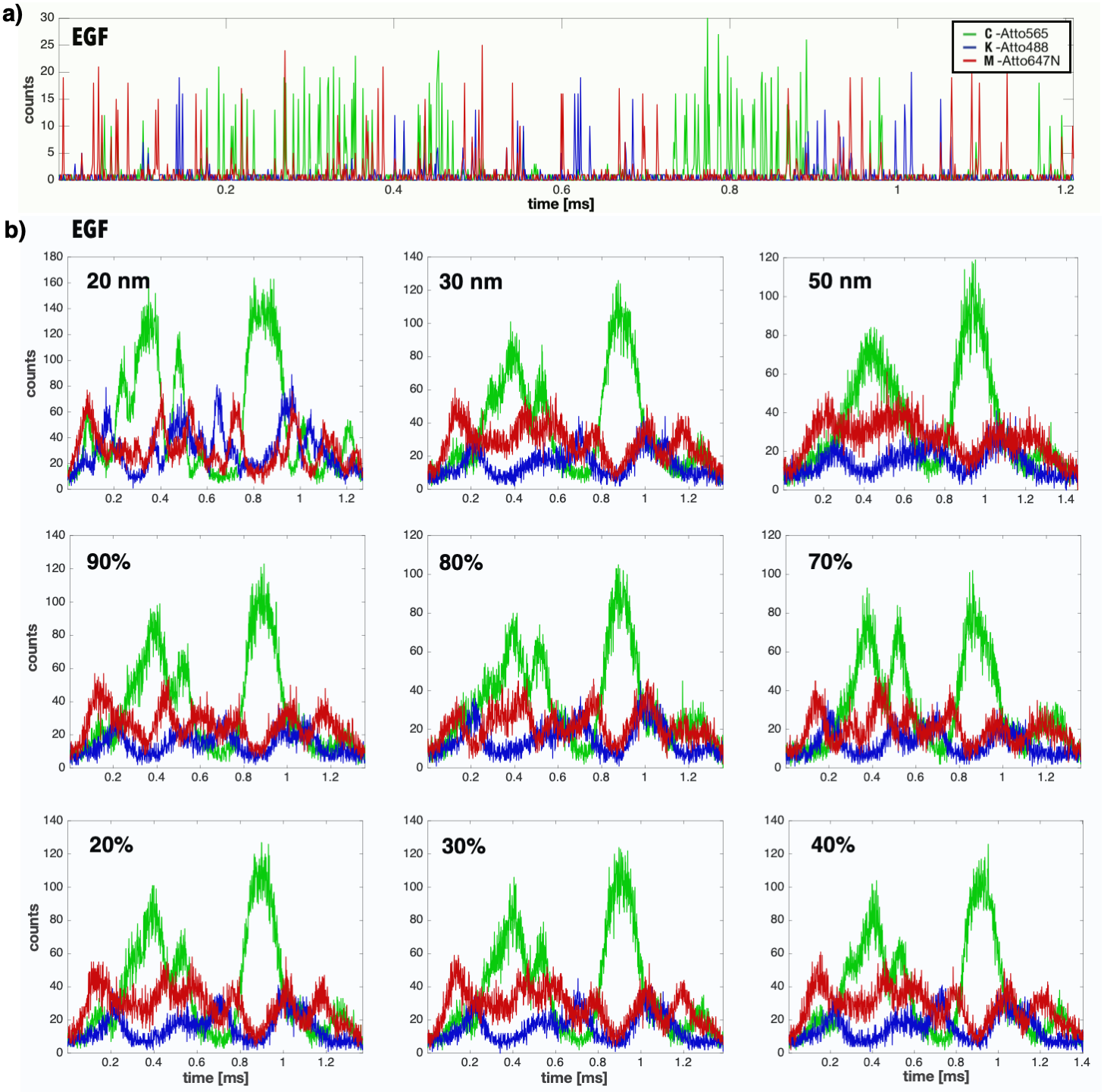

Supplement: S3 Fig — (a) optical signals simulated using a spatial resolution of 0.5nm and a labelling efficiency of 100%. (b) optical signals simulated using three distinct spatial resolutions: 10, 30 and 50nm (top), three distinct labeling efficiencies: 90%, 80% and 70% (middle), three velocity fluctuations: 20%, 30% and 40% of the mean translocation velocity v = 0.035 cm/s (bottom). (TIFF) [file pcbi.1007067.s004.tiff]

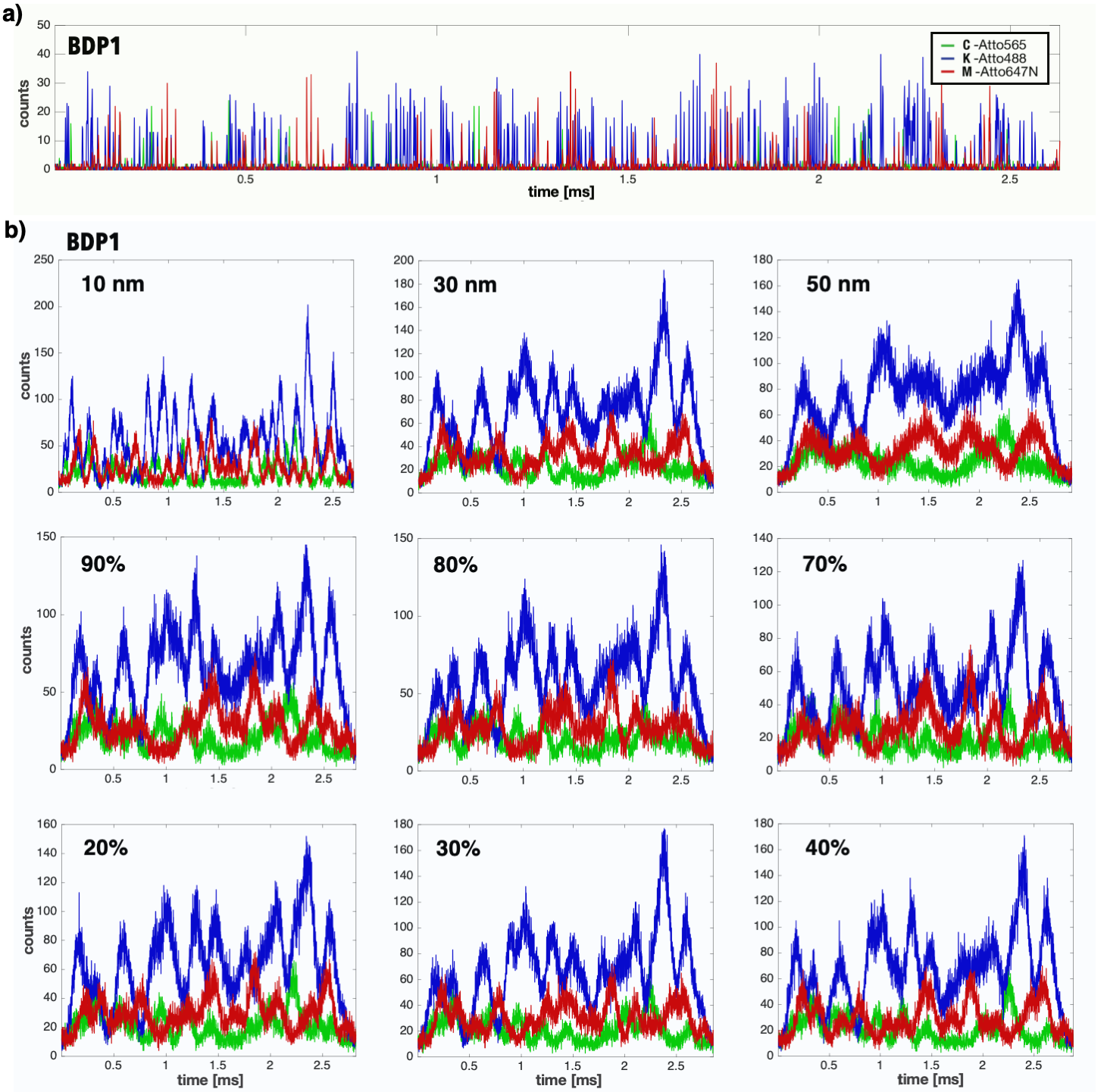

Supplement: S4 Fig — (a) optical signals simulated using a spatial resolution of 0.5nm and a labelling efficiency of 100%. (b) optical signals simulated using three distinct spatial resolutions: 10, 30 and 50nm (top), three distinct labeling efficiencies: 90%, 80% and 70% (middle), three velocity fluctuations: 20%, 30% and 40% of the mean translocation velocity v = 0.035 cm/s (bottom). (TIFF) [file pcbi.1007067.s005.tiff]

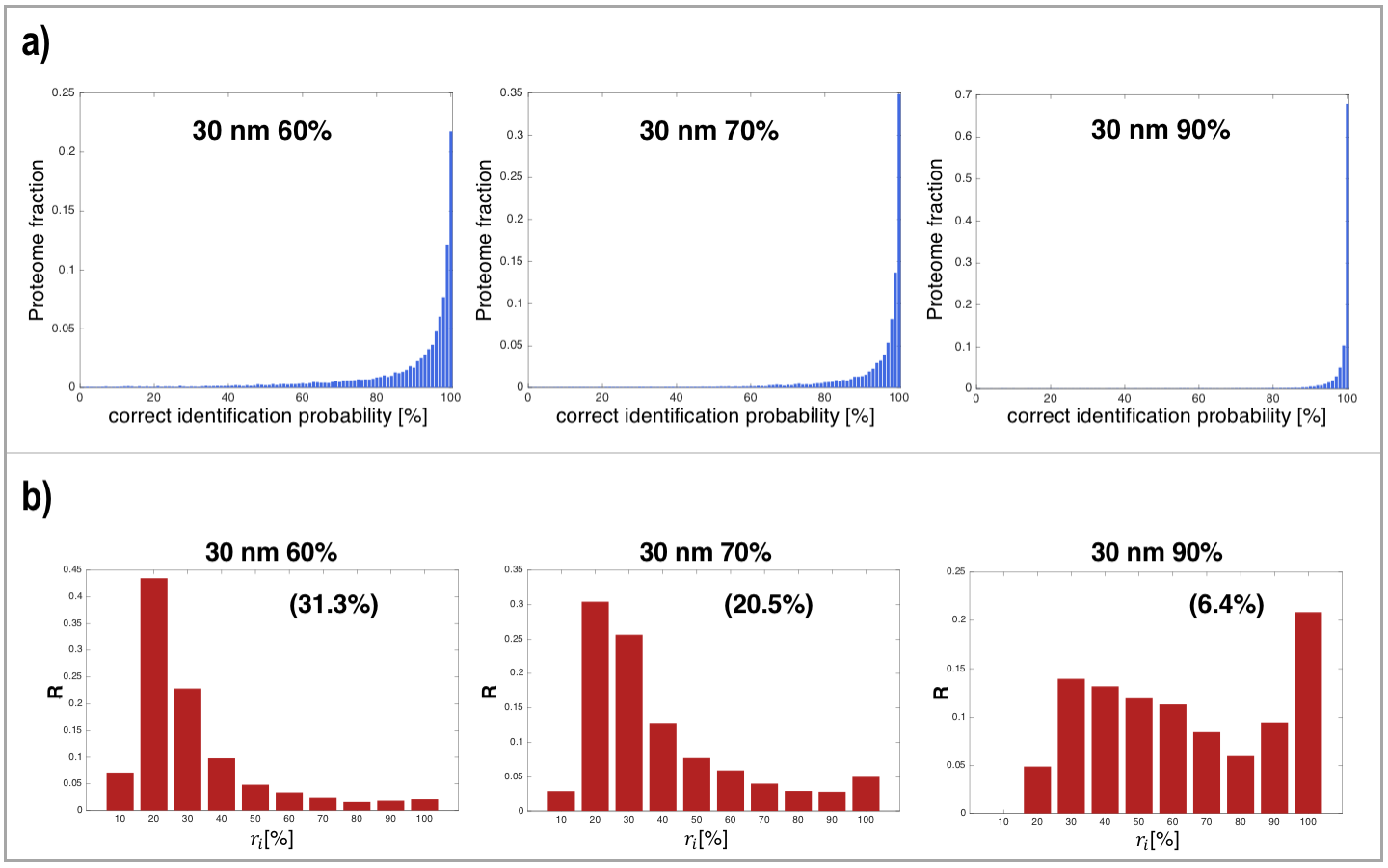

Supplement: S5 Fig — The fraction of the whole proteome that was correctly identified with probability p (a) and the degree of randomness in misclassification (b) were determined for 30nm and four labeling efficiencies (60, 70, and 90%; the remaining 80% as well as the CNN accuracy bar plot are shown in the article). The bin size was set to 1% in all histograms. The bin height of histograms in (b) is given by the fraction of mis-identified proteins R (i.e. proteins that had at least 10% of their events misclassified) at different ri (fraction of identical mismatch) intervals: ri = maxjnij/Ni for each protein i, where nij is the number of translocation events misidentified to protein j and Ni the total number of mis-classified translocation events. High is characteristic of a low degree of randomness, and vice-versa low of a high degree of randomness. The bin width–ri interval size–was set to 10%. The value in parentheses indicate the percentage of mis-identified proteins of a whole-proteome experiment. (TIFF) [file pcbi.1007067.s006.tiff]

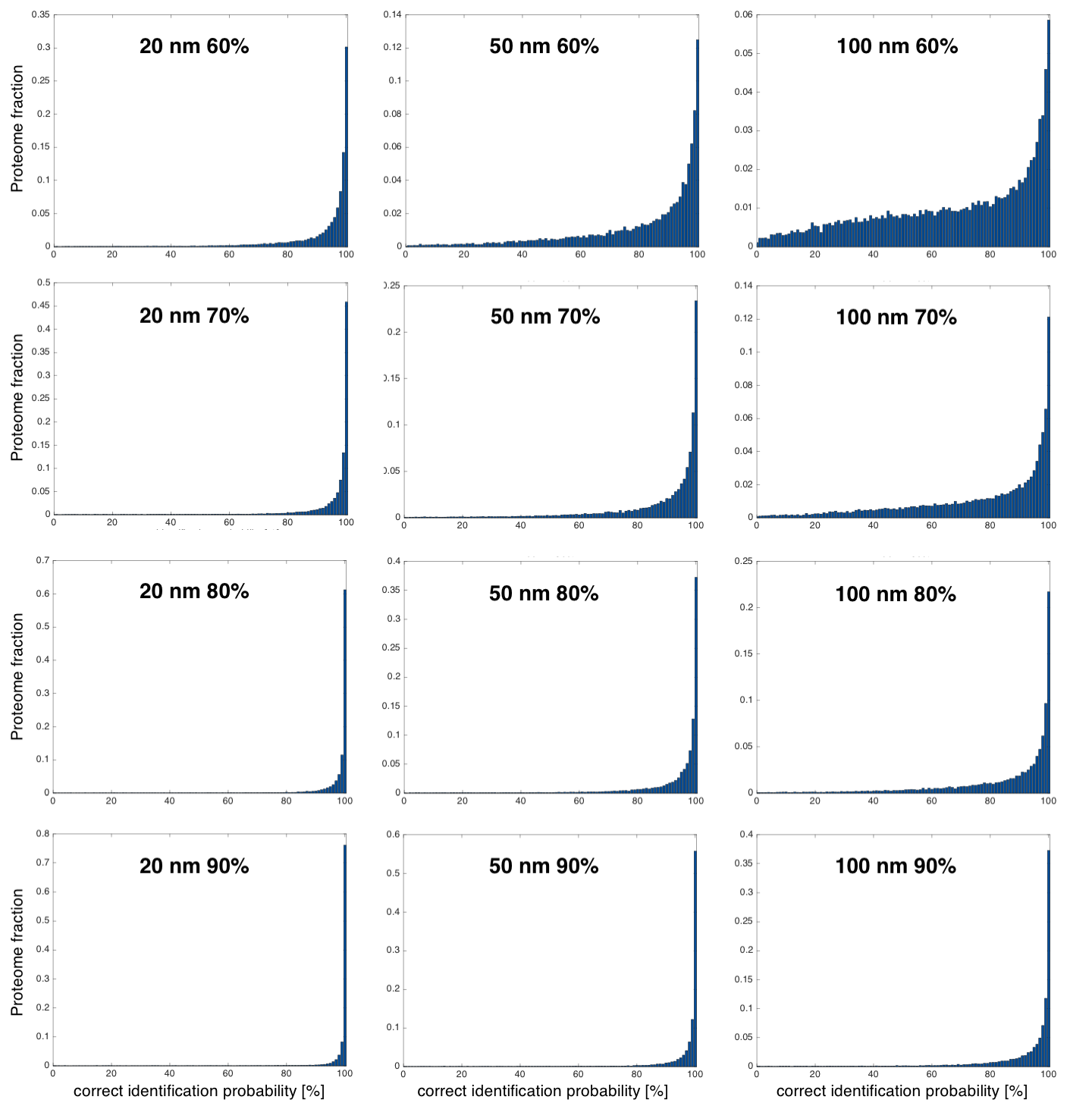

Supplement: S6 Fig — The fraction of the proteome that was correctly identified with probability p was determined for three spatial resolutions (20, 50 and 100nm; 30nm shown in article) and four labeling efficiencies (60, 70, 80 and 90%). The bin size was set to 1% in all histograms. (TIFF) [file pcbi.1007067.s007.tiff]

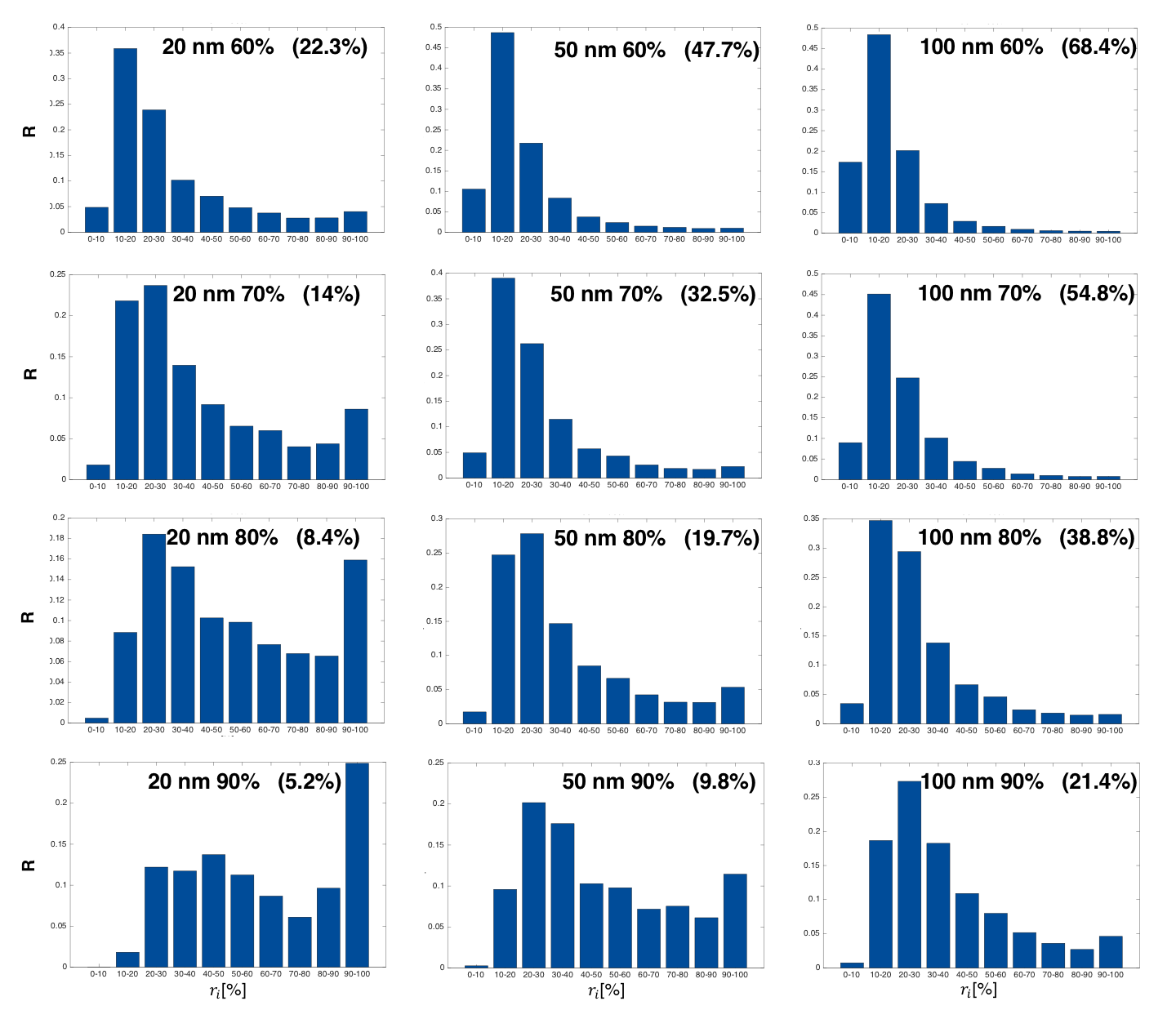

Supplement: S7 Fig — The bin height is given by the fraction of mis-identified proteins R (i.e. proteins that had at least 10% of their events misclassified) at different ri (fraction of identical mismatch) intervals: ri = maxjnij/Ni for each protein i, where nij is the number of translocation events misidentified to protein j and Ni the total number of mis-classified translocation events. High is characteristic of a low degree of randomness, and vice-versa low of a high degree of randomness. The bin width–ri interval size–was set to 10%. The value in parentheses indicate the percentage of mis-identified proteins of a whole-proteome experiment. The degree of randomness in misclassification was determined for three spatial resolutions (20, 50 and 100nm; 30nm shown in article) and four labeling efficiencies (60, 70, 80 and 90%). (TIFF) [file pcbi.1007067.s008.tiff]

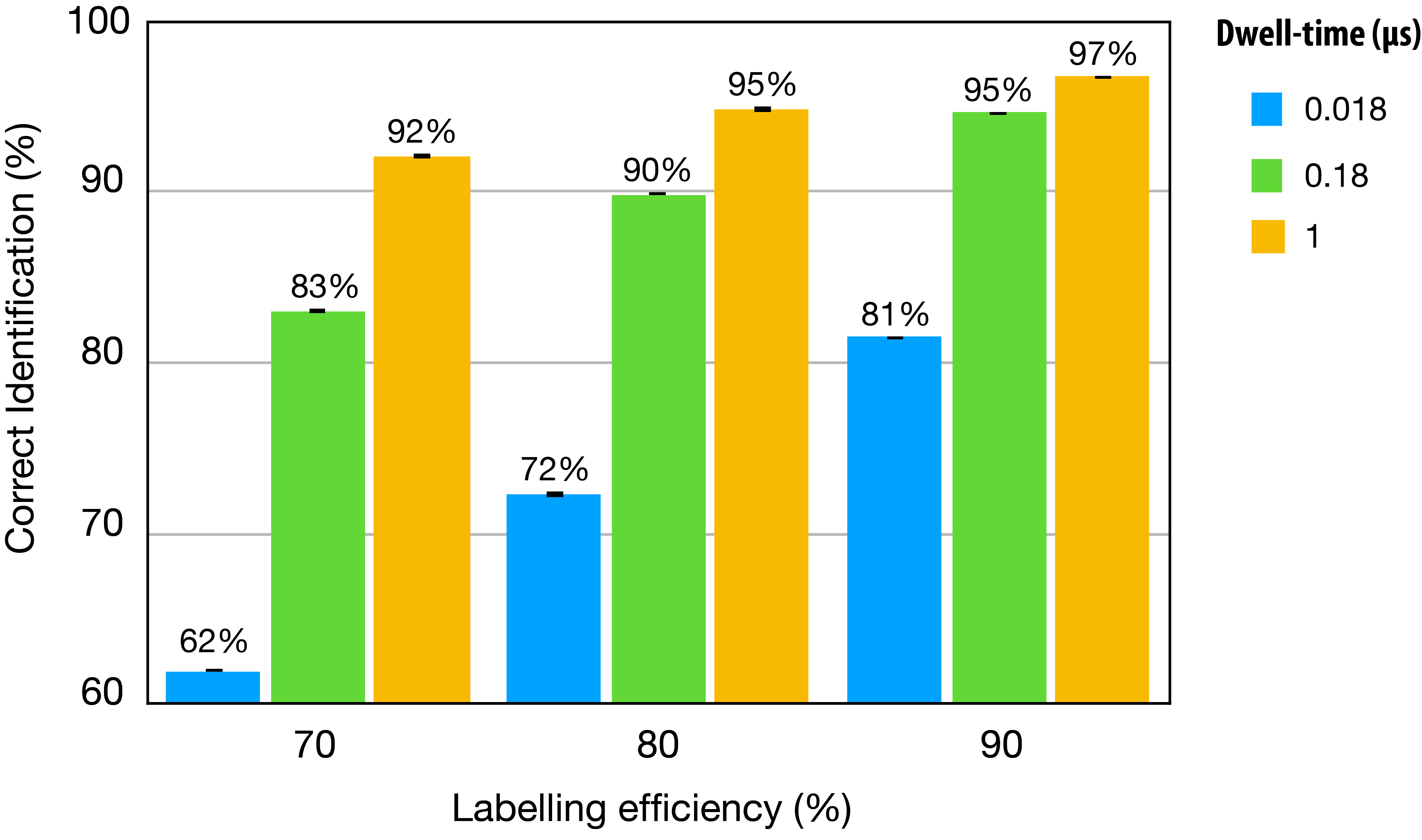

Supplement: S8 Fig — The spatial resolution was fixed to 30 nm and the dwell-time was defined as the time it took a peptide to translocate over the length of a single amino-acid. The corresponding translocation velocities are 2, 0.2 and 0.035 cm/s. The APD binning was set to 1 μs. The CNN classification was still robust to low labeling efficiency and realistic spatial and temporal resolutions, expected in real experiments. (TIF) [file pcbi.1007067.s009.tif]

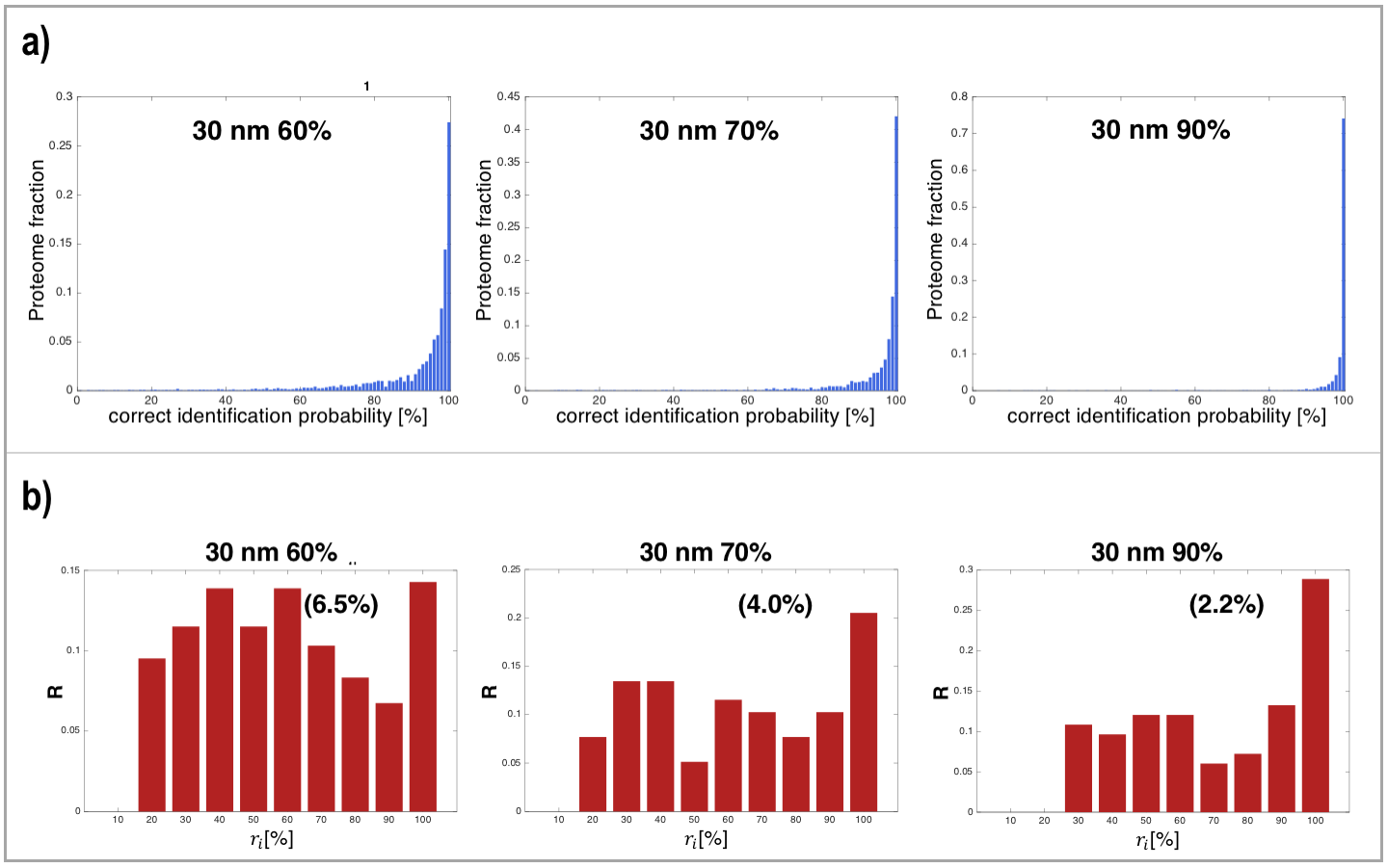

Supplement: S9 Fig — The fraction of the plasma proteome that was correctly identified with probability p (a) and the degree of randomness in misclassification (b) were determined for 30nm and four labeling efficiencies (60, 70, and 90%; the remaining 80% as well as the CNN accuracy bar plot are shown in the article). The bin size was set to 1% in all histograms. The bin height of histograms in (b) is given by the fraction of mis-identified proteins R (i.e. proteins that had at least 10% of their events misclassified) at different ri (fraction of identical mismatch) intervals: ri = maxjnij/Ni for each protein i, where nij is the number of translocation events misidentified to protein j and Ni the total number of mis-classified translocation events. High is characteristic of a low degree of randomness, and vice-versa low of a high degree of randomness. The bin width–ri interval size–was set to 10%. The value in parentheses indicate the percentage of mis-identified proteins of a plasma-proteome experiment. (TIFF) [file pcbi.1007067.s010.tiff]

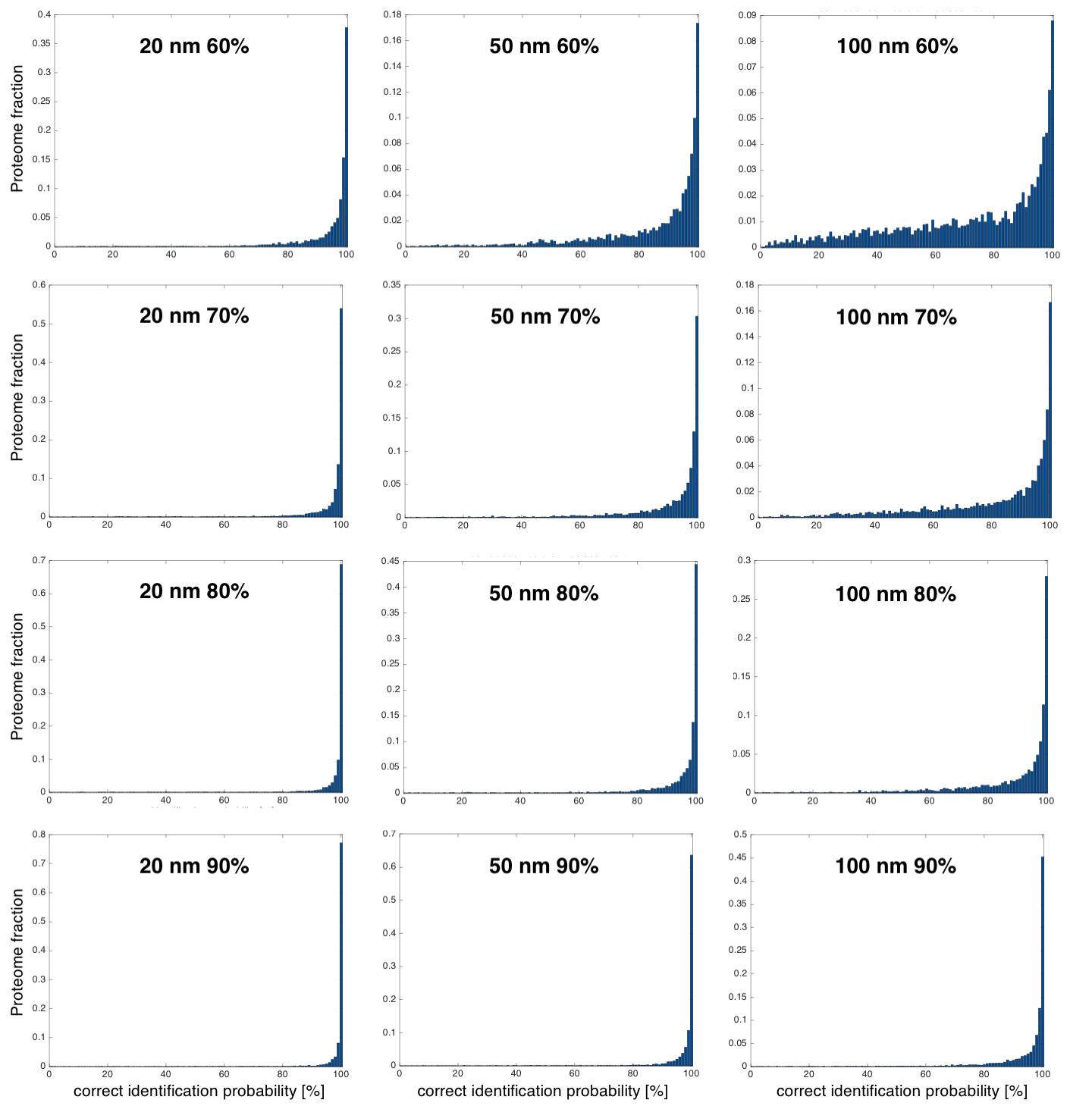

Supplement: S10 Fig — The fraction of the plasma proteome that was correctly identified with probability p was determined for three spatial resolutions (20, 50 and 100nm; 30nm shown in article) and four labeling efficiencies (60, 70, 80 and 90%). The bin size was set to 1% in all histograms. (TIFF) [file pcbi.1007067.s011.tiff]

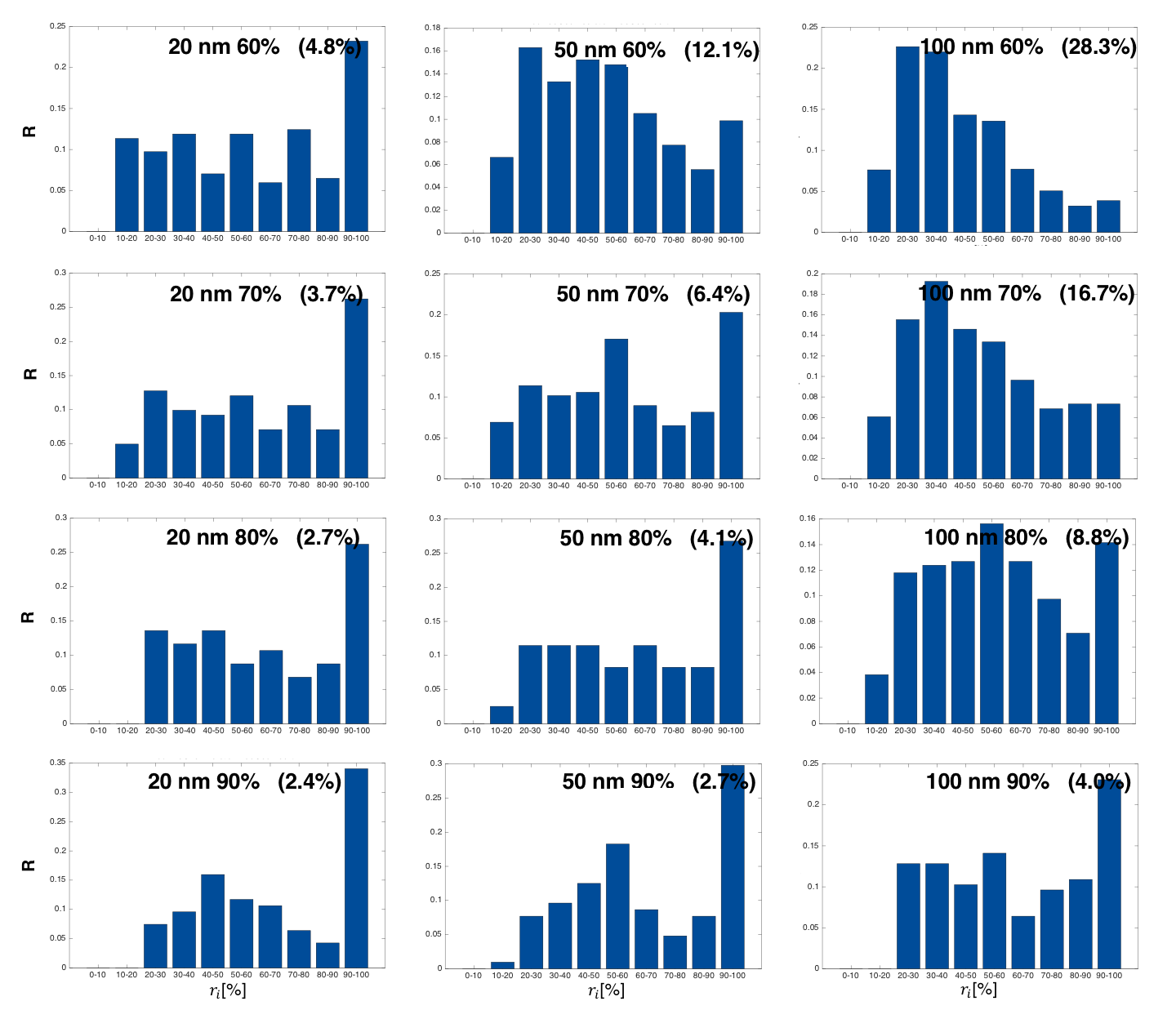

Supplement: S11 Fig — The bin height is given by the fraction of mis-identified proteins R (i.e. proteins that had at least 10% of their events misclassified) at different ri (fraction of identical mismatch) intervals: ri = maxjnij/Ni for each protein i, where nij is the number of translocation events misidentified to protein j and Ni the total number of mis-classified translocation events. High is characteristic of a low degree of randomness, and vice-versa low of a high degree of randomness. The bin width–ri interval size–was set to 10%. The value in parentheses indicate the percentage of mis-identified proteins of a plasma-proteome experiment. The degree of randomness in misclassification was determined for three spatial resolutions (20, 50 and 100nm; 30nm shown in article) and four labeling efficiencies (60, 70, 80 and 90%). (TIFF) [file pcbi.1007067.s012.tiff]

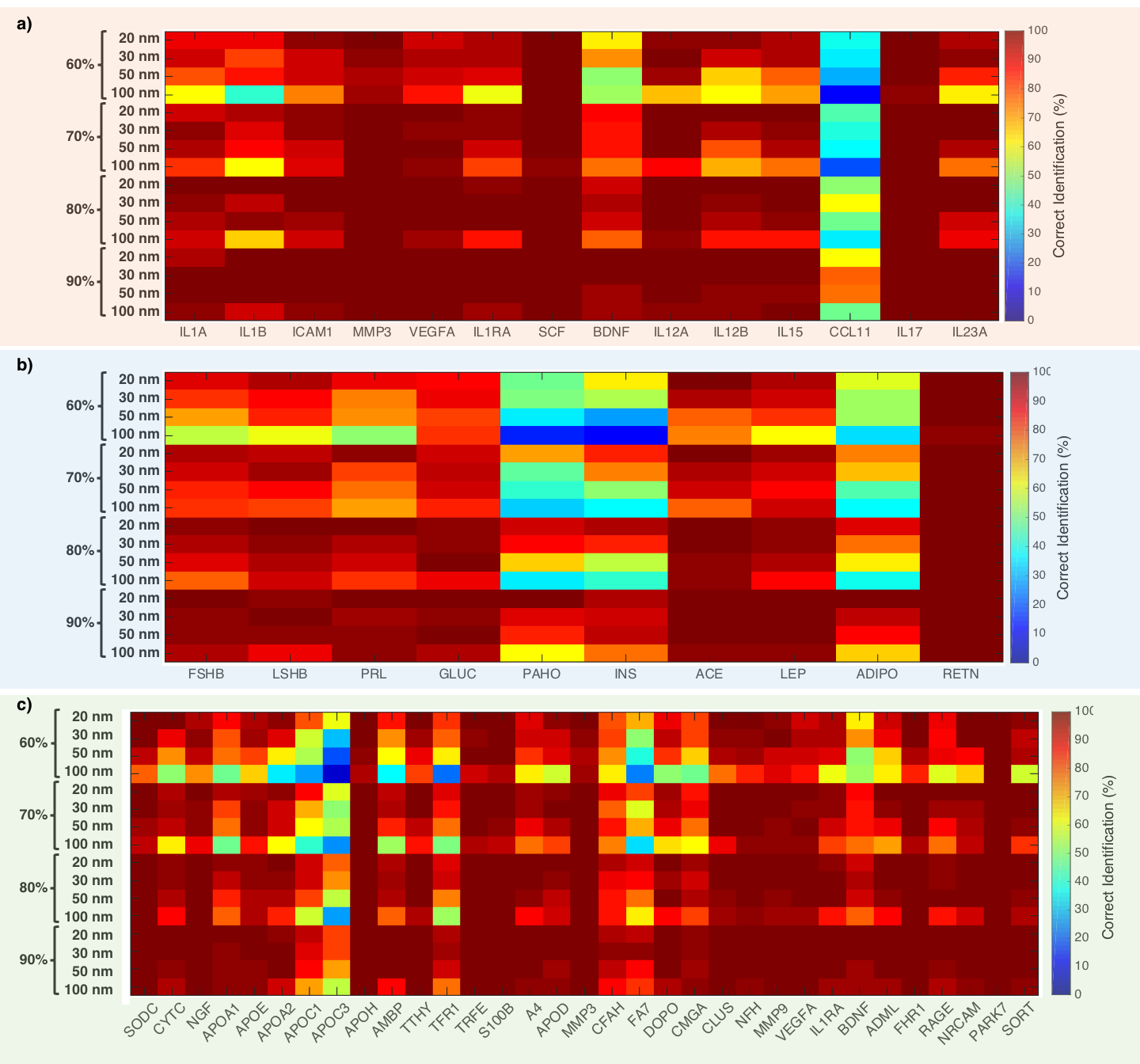

Supplement: S12 Fig — a) Whole-proteome CNN accuracy of the CytokineMAP B kit proteins for four spatial resolutions (20, 30, 50 and 100nm) and four labeling efficiencies (60, 70, 80 and 90%). b) Whole-proteome CNN accuracy of the MetabolicMAP kit proteins for four spatial resolutions (20, 30, 50 and 100nm) and four labeling efficiencies (60, 70, 80 and 90%). c) Whole-proteome CNN accuracy of the NeuroMAP A kit proteins and misclassification distribution for four spatial resolutions (20, 30, 50 and 100nm) and four labeling efficiencies (60, 70, 80 and 90%). (TIFF) [file pcbi.1007067.s013.tiff]

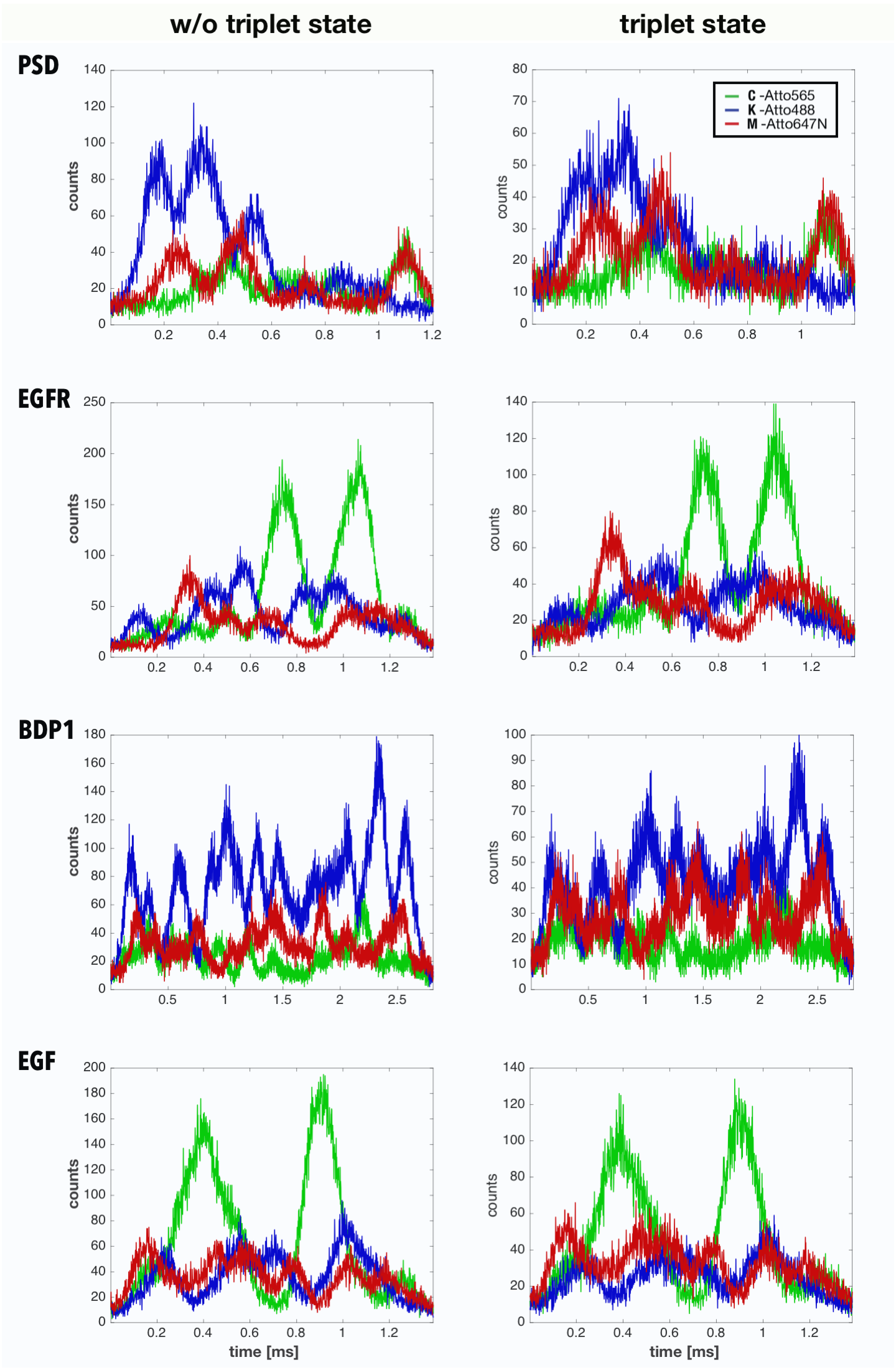

Supplement: S13 Fig — The spatial resolution and labeling efficiency were fixed in all cases to 30nm and 100%, respectively. Left column shows the simulated traces optical traces using a two-state (ground and excited) fluorophore model; right column using a three-state (ground, excited and triplet) model. Transition rates in between all states were determined according to the manufacturer (when available) and to published work (see Article). (TIFF) [file pcbi.1007067.s014.tiff]
